# Vegetables and Glycemic Index: Exploring Their Correlation and Health Implications

**DOI:** 10.3390/foods14213703

**Published:** 2025-10-29

**Authors:** Manish Kumar Singh, Hyeong Rok Yun, Jyotsna S. Ranbhise, Sunhee Han, Sung Soo Kim, Insug Kang

**Affiliations:** 1 Department of Biochemistry and Molecular Biology, School of Medicine, Kyung Hee University, Seoul 02447, Republic of Korea; manishbiochem@gmail.com (M.K.S.);; 2 Biomedical Science Institute, Kyung Hee University, Seoul 02447, Republic of Korea; 3 Department of Biomedical Science, Graduate School, Kyung Hee University, Seoul 02447, Republic of Korea

**Keywords:** carbohydrates, chronic disease, dietary fiber, glycemic index, vegetables

## Abstract

Background: Vegetables are consumed worldwide in various forms, including raw, as green leaves in salads, and as ingredients in a wide range of dishes, such as curries, sauces, and burgers. They are rich in carbohydrates and dietary fiber (DF), and also provide moderate amounts of protein, fat, oils, essential micronutrients, minerals, vitamins, and phytochemicals. Among their carbohydrate components, simple sugars such as monosaccharides/hexoses significantly influence postprandial blood glucose responses. The glycemic index (GI) is critical for managing chronic conditions, such as diabetes, obesity, hyperglycemia, and other metabolic diseases. The influence of individual carbohydrate fractions, such as hexoses, on GI and glycemic load (GL) has not been extensively investigated. Methods: This retrospective study analyzed the carbohydrates in vegetables (n = 65), focusing on hexoses and fibers, their carbohydrate-to-fiber ratio, and their effect on the GI and GL. Carbohydrate data were obtained from publicly accessible databases, including the U.S. Department of Agriculture (USDA), FooDB, European and Australian food databases, and PubMed. The study assessed total carbohydrates (TC), hexoses, dietary starch (DS), total sugars (TS), and DF, and examined their correlations with GI using regression analysis. Results: Our analysis revealed that fiber ratios are a more reliable predictor of GI than conventional net carbohydrate measures. Among the carbohydrates analyzed, TC exhibited the highest positive correlation with GI, both in absolute terms and when normalized to fiber, while TS showed a weak correlation. Among the ratios studied, TC demonstrated a stronger correlation with the GI, followed by DS. Conclusions: Comparative evaluation revealed that DF exerts a buffering effect on glycemic response (GR) and supports the use of fiber ratios as a more stable and intrinsic parameter for predicting GI than standard estimation methods. Traditional approaches that rely on net carbohydrates may overlook important factors affecting glycemic impact, particularly the buffering effects of dietary fiber. This study advocates for the incorporation of carbohydrate-to-fiber ratios into GI estimation models. Our research may help evaluate the carbohydrate content in vegetables for further in vitro and in vivo studies aimed at clarifying the mechanisms and validating these metrics in glycemic regulation.

## 1. Introduction

Vegetables are indispensable components of the human diet globally. They provide a diverse array of colors and are rich sources of starch, dietary fiber, vitamins, and bioactive compounds that contribute significantly to overall health and well-being. Multiple studies demonstrated that high intake of vegetables and fruits lowers the risk of chronic diseases. For over two decades, the World Health Organization (WHO) has recommended a minimum daily intake of 400 g of fruit and vegetables to prevent various metabolic diseases [1]. Global dietary guidelines generally recommend five portions of fruit and vegetables every day, with three of those portions being vegetables [2,3]. More recently, specific recommendations for daily vegetable intake have emerged, suggesting that a mean vegetable intake of 300 g is optimal for aligning both human and planetary health. A daily intake of 360 g is suggested to significantly reduce the global burden of disease. Insufficient vegetable intake is associated with adverse health outcomes and contributes to an estimated 1.5 million deaths annually from cardiovascular diseases (CVD) alone, with the greatest burden observed in low-and middle-income countries [4].

The most commonly consumed vegetables include crucifers, alliums, and solanaceous vegetables. Cruciferous vegetables, which include cabbage, broccoli, cauliflower, russels sprouts, kales, kalian, Chinese cabbage, turnip, rutabaga, radish, rocket, watercress, and mustard, provide the richest sources of glucosinolates in the human diet [5]. These cruciferous vegetables help protect against certain types of cancer, such as colon, rectal, and thyroid cancer [6]. Vegetables, such as garlic, onions, leeks, chives, and Welsh onions, belong to the allium family, are rich sources of thiosulfides, and have been shown to reduce the risk of chronic diseases. Solanaceous vegetables, such as tomatoes, potatoes, eggplants, and both sweet and hot peppers, are rich in lycopene, folate, and vitamins C, A, and E, playing a significant role in reducing the risk of CVD and various cancers. Eggplants and peppers are also excellent sources of fiber, minerals, especially iron, potassium, and vitamins, mainly vitamin C, vitamin B6, and vitamin K [7,8,9]. Dark green vegetables like spinach and cabbage provide at least 50% Dietary Reference Intake (DRI) for vitamin C and K. Legumes and the allium family bulbs provide 50% DRI for vitamin K. In comparison, tomatoes and other red fruits provide 50% DRI for vitamin C. The combination of green vegetables and legumes contains more than 25% of the DRI for folate, and the allium family bulbs contribute significant amounts of manganese and vitamin B6 [10]. Vegetable-based foods are typically low in calories because they contain a high-water content and a moderate quantity of fats. Nutritionally, they are rich in complex carbohydrates, fiber, minerals, and a wide range of micronutrients, including carotenoids, vitamins, minerals, and polyphenols. These bioactive compounds have therapeutic potential and may help regulate energy balance, reduce oxidative stress, and mitigate chronic pro-inflammatory states and metabolic disorders [11].

Plant polyphenols and their metabolites have been shown to have beneficial effects on blood pressure, as well as on markers of oxidation and inflammation. Additionally, polyphenols play a role in preventing or improving endothelial dysfunction through several mechanisms. These include reducing the expression of NADPH oxidase, enhancing the activity of antioxidant enzymes, exhibiting anti-inflammatory effects, increasing the bioavailability of nitric oxide, and inhibiting the oxidation of low-density lipoprotein (LDL) [12]. Other polyphenols, such as tyrosols, alkyl phenols, hydroxybenzaldehyde, furanocoumarins, and hydroxycoumarins, have also demonstrated positive effects on cardiovascular risk factors by enhancing antioxidant properties [13]. Significant associations have been found for lignans, flavanols, and hydroxybenzoic acids as well. Eggplants are rich in hydroxycinnamic acids, while spinach contains high levels of para-coumaric acid. These phenolic acids are typically found in conjugated forms, such as glycosides or esters [14]. Other studies have identified an inverse association between moderate intake of cruciferous vegetables and type 2 diabetes mellitus (T2DM) [15]. However, the exact biological mechanisms underlying the protective effects of vegetable consumption remain uncertain due to the variable quantities of micronutrients and other bioactive compounds present in various vegetables.

The GI of food is influenced by several factors, including the type of sugar, such as monosaccharides (like glucose or fructose), oligosaccharides (like sucrose or galactose), and complex carbohydrates (polysaccharides or starch). Additionally, the type of fiber, whether soluble or insoluble, affects blood glucose levels and insulin sensitivity [16]. Numerous studies have shown that both low-GI and high-GI foods significantly impact metabolism and blood sugar levels [17]. Although GI indicates a food’s potential to raise blood glucose levels, the overall blood glucose response to a meal is determined by both the quantity and quality (GI) of the carbohydrates consumed [18]. Research has revealed that a low-GI diet significantly reduces levels of fructosamine and hemoglobin A1c (HbA1c) in individuals with T2DM [19].

Vegetables are notable for containing non-starch components such as polysaccharides, which include cellulose, hemicellulose, gum, pectin, lignin, resistant dextrin, and resistant starch [20]. The distinct effects of non-starchy versus starchy vegetables on health remain largely unexplored. However, growing epidemiological evidence suggests that different types of vegetables may have varying health impacts. For example, a higher intake of non-starchy leafy vegetables is associated with weight loss, while increased consumption of starchy vegetables, such as potatoes, is linked to weight gain [21]. A cohort study in the USA found an inverse relationship between non-starchy vegetable intake and mortality, whereas starchy vegetables showed no significant effect [22]. Similarly, a cohort study in Japan indicated that higher intakes of non-starchy vegetables were associated with lower mortality rates [23]. The DF found in non-starchy vegetables has been shown to promote the production of short-chain fatty acids (SCFAs), improve insulin resistance, and assist in lowering cholesterol levels, while also fostering beneficial growth in gut microbiota [24]. Therefore, increased intake of non-starchy vegetables can positively influence gut health, reduce systemic inflammation, and enhance both microbial fermentation and SCFA production. Another study demonstrated that consuming starchy vegetables is inversely associated with fasting blood glucose levels [25], and the incidence of gestational diabetes mellitus among women in Tehran [26]. Moreover, several epidemiological studies reported an inverse association between non-starchy vegetable intake and overall mortality risk [27]. Interestingly, a survey noted that moderate consumption of starchy vegetables was also inversely associated with all-cause mortality in the Chinese population [28]. In conclusion, higher consumption of non-starchy vegetables compared to starchy vegetables is associated with greater health benefits, although both types demonstrate an inverse relationship with mortality risk [29]. Further investigation is warranted to validate these observations and gain a deeper understanding of the underlying mechanisms.

The biological mechanisms through which vegetable consumption protects against diabetes and its related biomarkers are not yet fully understood. However, preclinical studies have suggested several potential pathways. In vivo research suggests that increased vegetable intake may help reduce body weight, plasma glucose levels, and insulin resistance, thereby promoting glucose-insulin balance and lowering the risk of diabetes [30,31]. The synergistic effects of various nutrients and phytochemicals found in vegetables, particularly DF, are believed to significantly contribute to this protective effect. DF, especially abundant in vegetables, can delay gastric emptying and reduce postprandial glycemic responses, which may help reduce systemic inflammation and prevent weight gain [32,33]. Green leafy vegetables are particularly rich in essential nutrients and bioactive compounds such as β-carotene, lutein, folate, vitamin K1, and dietary nitrates [34,35]. Consuming these vegetables and their associated micronutrients has been linked to a decreased risk of developing diabetes [36,37,38]. Additionally, cruciferous vegetables, including broccoli, cabbage, cauliflower, radish, turnip, and wasabi, contain glucoraphanin, a precursor to sulforaphane (SFN), a compound known for its potent antioxidant and anti-inflammatory properties [39]. SFN has been shown to activate the nuclear factor erythroid 2-related factor 2 (Nrf2) pathway and inhibit hepatic gluconeogenesis by binding to key regulatory enzymes such as glucose-6-phosphatase (G6P) and phosphoenolpyruvate carboxykinase (PEPCK) [40]. These mechanisms underscore the potential of bioactive compounds derived from vegetables in the prevention and management of type 2 diabetes. To our knowledge, there have been limited studies that have explored the relationship between vegetable intake and markers of glucose tolerance and insulin sensitivity. This study investigates the analysis of vegetable carbohydrates and various hexoses, along with their ratios to dietary fiber, contributing to a reliable metric for calculating GI and GL. Furthermore, our study categorizes vegetables based on fiber-to-hexose ratios that have a significant association with GI. These findings may help design a more reliable system for classifying vegetables according to individual health status, aiding in the management and improvement of glucose tolerance and insulin sensitivity.

## 2. Materials and Methods

### 2.1. Vegetables-Based Sources of Carbohydrate Contents

A diverse variety of vegetables was selected for this study based on their availability and consumption patterns worldwide. The selection process depends on multiple factors, including leafy green vegetables, dietary fiber, vitamins, and nutrients, as well as health benefits, medicinal properties, and disease prevention potential. Different types of vegetables have varying specific nutritional values, such as carbohydrates (leguminous vegetables, sweet potatoes, potatoes, onions, garlic, and radishes) and proteins (peas, beans, and leafy vegetables). Since vegetables contribute essential macronutrients to the human diet, primarily carbohydrate polysaccharides including starch and fibers, their effect on blood glucose response and glycemic index was a key consideration. Subsequently, the selection was further refined to focus on individual hexoses, including glucose, fructose, and the disaccharide sucrose. Vegetables are also a good source of fiber and are available throughout the year. Vegetables are associated with various health outcomes, including high antioxidant content, low sugar levels, high fiber content, and prebiotic activity that supports gut microbiota. Vegetable components aid in weight management and decrease the risk of chronic disease. The nutritional composition of vegetables varies significantly depending on their genetic makeup and growing conditions, including environment and climatic conditions. Moreover, different types of vegetables can be consumed raw or cooked, boiled with various herbs and spices to enhance the taste and flavor. The boiled and raw vegetables are good to manage the sugar levels, including vitamins and minerals, soluble and insoluble fiber, resistant starch, total polyphenols, lignans, and antioxidant capacity, thereby contributing to overall health and well-being.

The nutritional value of vegetable components, particularly carbohydrates and dietary fiber, was sourced from various reputable public databases. These sources include the U.S. Department of Agriculture (USDA) Data Central, FooDB, Korean Food Composition Database (KFCD), European Food Safety Authority (EFSA), World Health Organization (WHO) Nutrient Data Portal, Food Standards Australia New Zealand, Mattilsynet, MyFoodData, NutrientOptimiser, NutritionValue.org, FoodStructure, PubMed, and other open-access food composition databases. For each vegetable, analytical values were extracted per 100 g. The TC (cumulative amount of all sugar types) and the TS (sum of glucose, fructose, and sucrose) values were compiled from multiple data sources. The specific data for the DS and the DF were also collected from the same resources. However, comprehensive data on the nutritional composition of vegetables remains limited.

### 2.2. Collection of GI and GL of Vegetables and Databases

The GI values for various vegetables were collected from the scientific literature and publicly available online sources [41,42,43]. Both the GI and the GL data for each vegetable were primarily sourced from reputable databases, such as the NHS Foundation Trust, which incorporates updated data following initial publication. The GI values were measured using either glucose or white bread as the reference food. Notably, when white bread was used as the reference in the original study, the GI value was multiplied by 0.7 to standardize it to glucose as the reference. The GL values were obtained for most of the grains by multiplying the amount of carbohydrate in a specified serving size (100 g) by the GI value of that food. The carbohydrate content was sourced from the reference studies or, when unavailable, from reliable food-composition tables [44,45]. Additional data sources included websites such as MyPlate.gov (USDA), GestationalDiabetic.com, UniversityHealthNews.com, NourishedByScience.com, Glycemic Index Guide, RedcliffeLabs.com, Newlifeobgyn.com, MSDManuals.com, Dasilvainstitute.com, the online Glycemic Index Database (www.gilisting.com), and the official GI website and databases (www.glycemicindex.com). The data were accessed between March 2025 and September 2025.

### 2.3. Assessment of Available Carbohydrate Content and Its Fiber Ratio

The vegetable samples were analyzed for their individual composition, including TC, individual hexoses, and dietary fiber. The available carbohydrate content was calculated using the following equation:

Available (net) carbohydrate (g) = Total carbohydrate (g) − Dietary fiber (g) [46,47,48].

This value represents the net carbohydrate content and serves as a standard reference for determining the GI of foods. A similar approach was applied to estimate the available total sugar and starch contents of individual vegetables, and these values were subsequently used to examine their correlation with GI and GL. Notably, vegetables contained significantly lower amounts of simple sugars, primarily hexose monosaccharides, compared with TC and DS. Consequently, applying the traditional subtraction method occasionally yielded negative values due to higher fiber content. These negative values do not indicate the presence of “negative sugar” in a given vegetable; rather, they reflect the mathematical outcome relative to dietary fiber content. Typically, vegetables exhibit higher amounts of complex carbohydrates (fiber and starch), resulting in positive values for most samples when compared with total sugar content. Nevertheless, a few vegetables still yield negative values due to their specific compositional profile (Table S1).

To address these anomalies, calculating the available carbohydrate content as a carbohydrates-to-fiber ratio eliminates negative values [16]. The TS amount represents the sum of glucose, fructose, and sucrose, whereas the TC content consists of all types of quantified carbohydrates [49,50,51,52]. The calculated carbohydrate-to-dietary fiber ratios (*g*/*g*) for the analyzed vegetables are presented in Table 1.

### 2.4. Venn Diagram

Venn diagrams were generated using the InteractiVenn online tool [53]. Advanced Interactive Venn Diagrams for Scientific Research were used for analysis via www.interactivenn.net. A maximum of six vegetable groups can be analyzed at a time. Separate Venn diagrams were constructed for each category: total carbohydrates, carbohydrate-to-fiber ratio, net carbohydrates, and net carbohydrate-to-fiber ratio.

### 2.5. Statistical Assessments

All statistical analyses were performed using Excel and GraphPad Prism (version 10.6.0). To identify the key components in the vegetable samples, Principal Component Analysis (PCA) was applied to reduce variable complexity among vegetables. Parallel analysis was used to evaluate the relationships between the variables by comparing the dataset eigenvalues with those obtained from random simulations. Subsequently, Multiple Linear Regression Analysis (MLRA) was applied to find the correlation between the vegetable components and the GI and the GL values. Pearson’s correlation coefficient (r) was calculated to measure association strength, with *p*-values determined using two-tailed tests at a 95% confidence level. Additionally, Pearson’s linear correlation analysis was performed to find the significant correlation between the GI, the GL, and individual vegetable components. The correlation coefficient (R) and regression line were derived from the best-fit values for the slope and intercept. Correlation strength was classified as weak for statistically significant R values below 0.4, moderate (0.4–0.6), and strong for values above 0.6. Biostatistical methods were used to calculate GI and carbohydrate content, and results were expressed as mean ± standard deviation (SD). Statistical significance was set at *p* < 0.05, based on two-tailed tests and one-way ANOVA at a 95% confidence level.

## 3. Results

The primary objective of this study was to focus on the impact of available carbohydrates, specifically TC, monosaccharides (glucose and fructose), TS, DS, and DF, on the GI of various vegetables. Vegetables are staple components of European and Asian diets, and are consumed in diverse forms, including fresh, blended, and lightly cooked or boiled preparations. Green vegetables, in particular, are rich in dietary fiber and bioactive compounds such as phenolics, nitrates, and phytosterols, which contribute to their nutritional value and health-promoting properties. Variations in fiber quantity and composition among vegetable types are key factors associated with improved health outcomes and reduced chronic disease risk. In this study, the carbohydrate profiles of individual vegetables were categorized as TC (g), hexoses (glucose, fructose) (g), DF (g), and TS (g) as shown in Table 1. Although vegetables contain simple sugars such as glucose, fructose, and sucrose, their quantities are relatively low and do not significantly elevate postprandial blood glucose levels. Consequently, these sugars are considered negative regulators of blood glucose response. According to the WHO, a daily intake of at least 400 g of vegetables is associated with positive health outcomes. This study further categorized vegetables based on their potential impact on both GI and GL. The estimated ratios are represented in Table 1.

Carbohydrate-to-fiber ratios have been associated with lower risk for T2DM and CVD [46,54]. The carbohydrate quality index (CQI) accounts for DF, GI, TC:DF ratio, and solid-to-liquid carbohydrates ratio [55], and has been proposed as a broader measure of carbohydrate quality. However, comparison between CQI and TC:DF ratios in predicting waist circumference changes indicates that the TC:DF ratio may be a stronger predictor. Recent evidence also suggests that this ratio provides more meaningful insights than simple absolute differences between TC and fiber, as used in standard analytical methods [16]. The TC:DF ratio more directly reflects the physiological interaction between carbohydrate and fiber, illustrating how fiber influences digestion rate, insulin response, glycemic regulation, and related metabolic outcomes. Unlike absolute carbohydrate values, the TC:DF ratio highlights the moderating role in the metabolic effects of vegetable-derived carbohydrates, enabling identification of vegetables that minimize postprandial glucose spikes while maintaining high nutritional value and a low glycemic index. In this study, the carbohydrate components, including TC, hexoses, DS, TS, and DF, of sixty-five commonly available and consumed vegetables were analyzed to evaluate their correlation with GI and GL. These variables were further examined to determine which most strongly predicted GR. Net available carbohydrate and corresponding fiber ratios were also quantified using standard analytical methods and are presented in Table S1. This analysis aims to identify the carbohydrate metrics that best capture the glycemic impact of vegetables, offering practical indicators for assessing carbohydrate quality and metabolic relevance.

**Figure 1:**
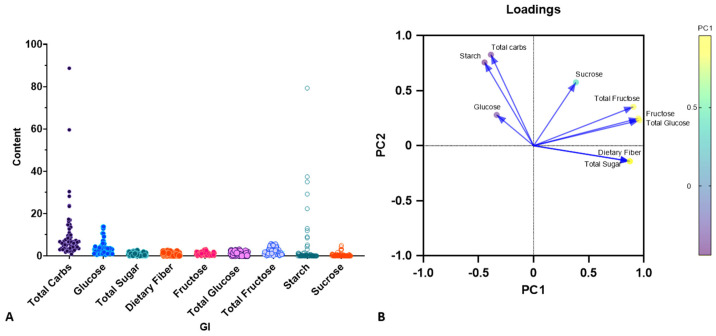
PCA of carbohydrate components (TC, TS, DS, and DF) and GI in various vegetables (n = 65). The correlation plot illustrates the relationship between the GI and carbohydrate contents. (**A**). Scatter plots show correlations between individual carbohydrate types and GI, where each data point represents a single vegetable, with carbohydrate content on the *Y*-axis and GI on the *X*-axis. (**B**). PCA biplot displaying loading vectors and principal component (PC) scores.

The Principal Component Analysis was conducted to identify the key contributors to variability in the dataset affecting GI. The PCA results revealed that PC1 accounted for 53.04% of the total variance, the highest proportion among the nine evaluated components. This suggests that PC1 had the most dominant influence. Variables with high positive loadings on PC1 included TS, DF, fructose, total glucose (TG), and total fructose (TF), all constituents primarily associated with sugar and fiber content. The PC2 explained an additional 21.60% of the variance and was primarily associated with TC, starch, and sucrose (Table 2). Combined, PC1 and PC2 accounted for 74.64% of the total variance, exceeding the 95th percentile threshold determined through parallel analysis. PC1 appeared to differentiate vegetables high in simple sugar from those richer in TS, DF, fructose, TG, TF, and sucrose, while PC2 reflected variability associated with TC, glucose, fructose, TG, TF, starch, and sucrose content (Figure 1A,B). Notably, the loading scores of TC (0.827) and DS (0.757) on PC2 were higher than on PC1, indicating their greater relevance in PC2. A strong correlation was also observed between TC and DS, as evidenced by the clustering pattern in the correlation matrix (Table 2). The loading scores of TS, DF, fructose, glucose, and TF on PC1 further underscore the close relationship among these variables across vegetable samples. These findings suggest that TC and DS play critical roles in modulating both GI and GL in vegetable-enriched diets. The insights gained from this analysis provide a valuable framework for identifying vegetables with favorable nutritional profiles for glycemic control.

**Figure 2:**
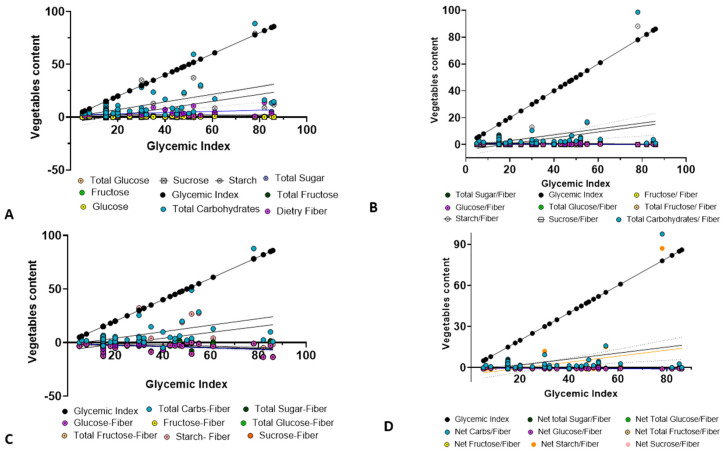
MLRA of carbohydrate components in vegetables (n = 65) in relation to GI. (**A**) MLRA between the carbohydrate contents of vegetables versus GI. (**B**) MLRA of carbohydrate-to-fiber ratios versus GI. (**C**) Correlation between available carbohydrate contents and GI. (**D**) Correlation between available carbohydrate-to-fiber ratios and GI. Each data point represents an individual vegetable, with carbohydrate content on the *Y*-axis and GI on the *X*-axis.

Further correlation analysis was performed to evaluate the relationship between individual vegetable components and both GI and GL using multiple linear regression analysis (MLRA). The results revealed that TC and DS exhibited a strong positive correlation with GI. In particular, TC demonstrated a statistically significant correlation, with a high Pearson’s correlation coefficient r- and *p*-values (r = 0.5307; *p* < 0.0001), as did DS (r = 0.5276; *p* < 0.0001). In contrast, glucose, fructose, and DF showed weaker correlations, with lower r- values and limited statistical significance (Figure 2A; Table 3). Among all variables analyzed, TC emerged as the strongest and most consistent positive predictor of GI, followed closely by DS. The data points corresponding to TC and DS were closely clustered around the GI regression line, indicating a strong linear relationship. In contrast, the data points for TS and DF were more widely dispersed and often clustered near the *x*-axis, suggesting a weaker correlation with GI.

When analyzing carbohydrate-to-fiber ratios, both the TC-to-DF and DS-to-DF ratios showed statistically significant correlations with GI, whereas the TS-to-DF ratio did not (Figure 2B). The corresponding Pearson’s r-values and *p*-values are summarized in Table 3. Notably, the fiber ratios demonstrated a stronger correlation with GI compared to individual carbohydrate components; however, the associated *p*-values were lower than those observed for individual components and GI (Table 3).

Further correlation analysis between available carbohydrate content and GI, conducted separately for individual hexoses, confirmed these observations. To assess the impact of vegetable-derived carbohydrates, particularly available (net) carbohydrates, on GI and GL, we employed a conventional method to measure the available carbohydrate content in various vegetable samples. This method is commonly used to estimate GI based on the available carbohydrate content (g). As anticipated and described in the Methods section, negative values were observed for available carbohydrates, particularly for hexose sugars such as glucose and fructose, as shown in Table S1. This outcome likely reflects the high fiber and macronutrient content, as well as the low sugar levels, which are typical characteristics of many vegetables. These results suggest that individual monosaccharides may exert a minimal influence on the overall GR compared to TC and more complex carbohydrates. We evaluated the correlation between available carbohydrate content and GI separately for individual hexoses. As expected, we found a significant correlation between TC and DS with the GI. However, the individual monosaccharides exhibited only weak association with the GI (Figure 2C). A summary of Pearson’s correlation coefficients (r) and the corresponding *p*-values for these analyses is summarized in Table 4. This finding reinforces the limited role of glucose, fructose, and sucrose from vegetables in elevating blood glucose levels.

Additionally, the ratios of vegetable-derived available carbohydrate-to-fiber further supported a strong relationship between available TC and DS with GI. In contrast, TG and TS again failed to demonstrate significant correlations (Figure 2D; Table 4). Collectively, these findings indicate that TC and DS are key determinants of GI in mixed vegetable diets. Conversely, individual hexose sugars such as glucose and fructose, either free or dissociated from complex sugar, appear to have minimal influence on GR. These findings suggest that the glycemic impact of vegetables is primarily driven by their complex carbohydrate content rather than by simple sugars.

The quantity and quality of carbohydrates remain central and sometimes controversial factors in determining the GI of foods or in mixed meals. Although GI is traditionally considered an intrinsic property of individual foods, remaining relatively stable when consumed with mixed foods, its ability to accurately predict postprandial GR in mixed foods has been questioned. Several approaches have been proposed to estimate GI in the context of mixed meals, but these methods vary in their assumptions and accuracy. GL offers an alternative framework and has been widely used in epidemiological studies to evaluate the glycemic impact of diets [56]. Unlike GI, which only accounts for carbohydrate quality, GL incorporates both the GI of a food and the quantity consumed per serving (g). Thus, it provides a more comprehensive estimate of cumulative GR across different intake events. However, due to the wide variability in the GI of staple carbohydrate foods and inaccuracies in dietary reporting methods such as food frequency questionnaires, combining GL with dietary intake data can introduce potential errors in large-scale cohort studies.

In this study, we extended our analysis by evaluating the correlations among various carbohydrate components in vegetables and GL using MLRA. Our results revealed that TC, DS, and DF were significantly associated with GL (Figure S1A). Among these, TC exhibited a weak positive correlation with GL (r = 0.2937; *p* = 0.0176), whereas DF showed a stronger correlation (r = 0.5017; *p* < 0.0001), indicating a more robust association compared to TC. In contrast, TS and individual hexoses (glucose and fructose) displayed relatively weak correlations, as evidenced by lower Pearson’s *r* values and higher *p*-values (Table S2). Data points for DF, TC, and DS were tightly clustered along the GL regression line, while those for TS and hexoses were more dispersed and clustered near the *X*-axis, suggesting weaker associations. When carbohydrate-to-fiber ratios were analyzed, neither the TC-to-DF nor the hexose-to-DF ratios demonstrated significant correlations with GL (Figure S1B and Table S2). We further examined the net carbohydrate content, using conventional standard methods. Interestingly, this analysis revealed an inverse trend, i.e., individual hexoses were significantly correlated with GL when expressed as net carbohydrates, whereas TC and DS were not. The corresponding Pearson’s r and *p*-values are summarized in Figure S1C and Table S3.

Further, we continued to extend these results. The ratios of net carbohydrate content to DF were examined. Interestingly, this analysis revealed non-significant correlations between all assessed components and GL. Most data points from the vegetable dataset were clustered near the origin of the regression plot. Among the evaluated ratios, neither hexose sugars nor complex carbohydrates demonstrated significant intercorrelations. Moreover, TC and DF alone were unable to reach a significant association with GL, as reflected by their respective correlation coefficient and *p*-values (Table S3), with data points remaining close to the *x*-axis (Figure S1D). These results suggest that TC, DS, and DF are not reliable predictors of GL in vegetables, nor are individual hexose sugars. Although hexoses reached statistical significance in some analyses, their physiological contribution to GL appears minimal. Notably, vegetables with higher TC and DS contents displayed stronger associations with GL when expressed as ratios to fiber; however, this trend was not consistent across all measures. Therefore, it is likely that non-carbohydrate constituents such as proteins, fat, or phenolic compounds play a more substantial and previously underappreciated role in modulating the GR to vegetables. Future research should consider these additional factors to more accurately assess the determinants of GL in vegetable-based diets.

**Figure 3:**
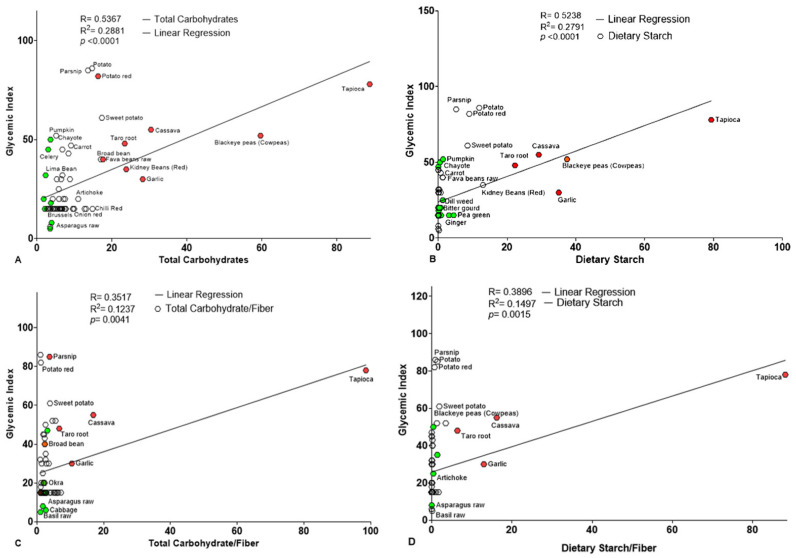
Correlation plot illustrating the relationships between GI and carbohydrate contents, as well as carbohydrate-to-DF ratios, in vegetables (n = 65). (**A**) Correlations between the GI versus TC (**B**), Correlations between the GI versus DS (**C**), Correlations between GI versus TC-to-DF ratio (**D**), Correlations between GI versus DS-to-DF ratio. In all panels, each data point represents a distinct vegetable sample, with GI values on the *Y*-axis and carbohydrate metrics on the *X*-axis. Solid lines indicate linear regression trends, highlighting the strength and direction of associations. Selected data points are labeled in green (low) and orange (high) for emphasis and identification.

Further investigations were conducted to validate the findings, examining individual hexoses and TC separately using Pearson’s linear regression. The results showed a strong positive association between TC with the GI, with a significant Pearson’s correlation coefficient (r = 0.5367 and *p* < 0.0001). Similarly, DS also exhibited a significant correlation with the GI (r = 0.5238 and *p* < 0.0001) (Figure 3A,B and Table 5). Graphical analysis revealed that several vegetables, such as ash gourd, Brussels sprouts, courgette, celery, cabbage, lettuce, rocket, lima bean, and radish, had low TC and starch contents, clustering near the regression line and *y*-axis in both TC and DS analyses. Additionally, this observation suggested that arugula, celery, lettuce, mature spinach, and kale contribute a limited role to trigger spontaneous postprandial GR. In contrast, vegetables such as green peas, broad beans, sweet potatoes, taro root, cowpeas, and tapioca contained higher amounts of TC, glucose, fructose, and starch, suggesting a higher potential to significantly increase the postprandial blood sugar level and, ultimately, the GI. These findings imply that incorporating an appropriate proportion of low-GI vegetables and minimizing the amount of high-GI vegetables into the diet may play a significant role in improving glycemic regulation, both GI and GL, and reducing the risk of chronic diseases.

Further analysis of the fiber ratios to hexoses showed enhanced correlations with GI. The TC-to-DF and DS-to-DF ratios were significantly correlated with GI, though with slightly lower Pearson’s r- values and *p*-values compared to the TC alone (r = 0.3517 and *p* = 0.0041; and r = 0.3869 and *p* = 0.0015 for DS) (Figure 3C,D and Table 6). Conversely, TS and TG showed a weak, negative, and non-significant correlation with GI, both independently and when expressed relative to fiber. This suggests that individual sugar, whether considered alone or in combination, may not be a strong determinant of GI compared to total carbohydrate content. These findings challenge the view that simple sugars are key contributors to GI and GL, indicating that more complex carbohydrates may play a larger role.

**Figure 4:**
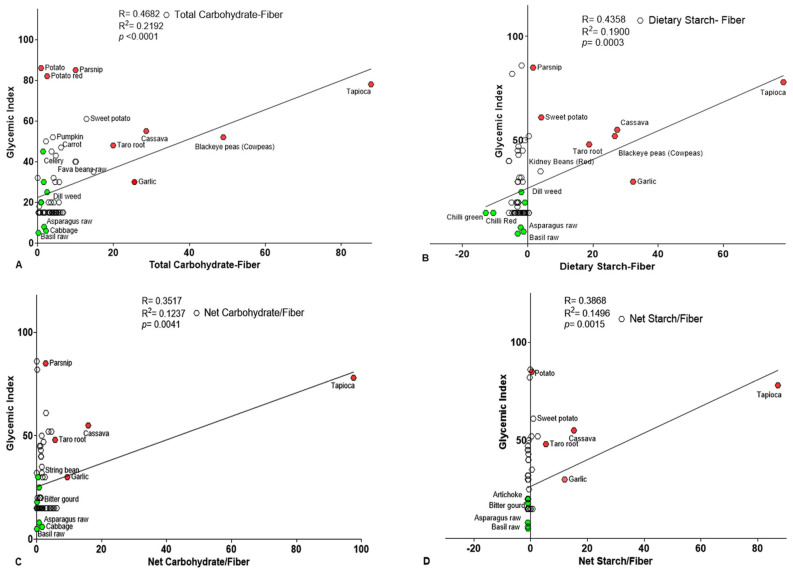
The correlation plot illustrates the relationship between the GI and vegetable-derived available carbohydrate content as well as net carbohydrate-to-DF ratios in vegetables (n = 65). (**A**) Correlations between GI versus available TC. (**B**) Correlations between the GI versus available DS (**C**), Correlations between GI versus available TC-to-DF ratio. (**D**) Correlations between GI versus available DS-to-DF ratio. In all panels, each data point represents a distinct vegetable sample, with GI values on the *Y*-axis and carbohydrate metrics on the *X*-axis. Solid lines indicate linear regression trends, reflecting the strength and direction of associations. Selected data points are labeled in green (low content) and red (high content) for emphasis and identification.

Additional analyses using conventional methods revealed that net carbohydrate content was more strongly correlated with GI. Specifically, net TC and net DS demonstrated robust positive correlations with GI, reinforcing the importance of these two components in the GI determination, particularly in a vegetable diet. The corresponding Pearson’s coefficient r- and *p*-values (r = 0.4682 and *p* < 0.0001 for TC and r = 0.4358, *p* = 0.0003 for DS) were, respectively (Figure 4A,B and Table 7). In contrast, glucose, fructose, and TS exhibited a significant negative correlation with GI (Table 7). Notably, TG, fructose, and sucrose displayed a weaker inverse correlation, reinforcing the negative impact of simple sugars on GI. These results r- and *p*-values are represented in Table 7, indicating a consistent inverse relationship between GI and simple sugars (glucose, fructose, sucrose), and a positive association with complex carbohydrates (TC and DS). This may reflect a metabolic or compositional shift favoring polysaccharide accumulation over monosaccharide content in determining GI values. Graphical analysis further supported this, with most vegetables showing low levels of individual hexoses dispersed towards the negative portion of the *x*-axis, while vegetables with higher TC and DS clustered near the regression line on the right side of the *x*-axis. Vegetables such as fava beans, watercress, Brussels sprouts, green chili, raw basil, arugula, kale, bitter gourd, parsley, chicory, string beans, and red potatoes consistently exhibited low hexose content. Conversely, raw fennel, green bell pepper, pumpkin, orange and yellow bell peppers, red bell pepper, white onion, sweet potato, and parsnip showed higher sugar content. Therefore, they were positioned on the positive *x*-axis, while those with low sugar content were either positioned near the *x*-*y* axis or on the negative portion of the *x*-axis.

Additionally, the net carbohydrate-to-fiber ratio also showed a significant correlation with net TC and net DS. Data analysis revealed significant Pearson’s r and *p* value for both components with r = 0.3517; *p* = 0.0041 and r = 0.3836 and *p* = 0.0015, respectively (Figure 4C,D and Table 8). Conversely, the net TS displayed a negative and non-significant relationship in either case. Apart from TS, individual hexoses glucose and fructose also displayed statistically significant negative correlation with GI. While TG and TF showed weak negative correlation, with *p*-values near the 0.05 significance threshold (Table 8). The fiber ratios to net available content revealed a divergence in the relationship between simple sugar, particularly glucose and fructose, and complex carbohydrates (TC and DS). Specifically, hexose sugar contents tend to decrease, and complex carbohydrate contents increase in association with the variable examined. This pattern may reflect shifts in carbohydrate partitioning, potentially affected by metabolic, genetic, or environmental factors. The graphs showed that the data points were dispersed on both sides of the x-axis in the case of hexoses (graphs are not included), while in the case of complex sugars, TC and DS, the data points are clustered near the *y*-axis and near the regression line (Figure 4C,D). A comparative analysis of net TC content (g) across the evaluated vegetables revealed a distinct categorization based on available sugar content. Vegetables such as green chili (0.01 g), parsley (0.07 g), kale (0.08 g), arugula (rocket) (0.13 g), chicory (0.18 g), red chili (0.40 g), and mature spinach (0.65 g) were classified as low-sugar vegetables. In contrast, pumpkin (3.64 g), white onion (5.40 g), and cucumber (6.20 g) exhibited relatively higher sugar content among the analyzed vegetable carbohydrates. These findings underscore the variability in carbohydrate composition across vegetable types. Further analysis is warranted to elucidate the relationship between these compositional differences and GR.

Numerous studies established GL as a key factor in regulating postprandial blood glucose levels and serve as a significant risk factor for chronic diseases such as T2DM and CVD. Therefore, further assessment was conducted to examine the impact of vegetable sugars on GL. The results demonstrated varied correlation across hexose sugar and GL. Linear regression analysis revealed a moderate but statistically significant correlation between TC and DS with GL, with Pearson’s r- and *p*-values (r = 0.2937 and *p* = 0.0176 for TC, and r = 0.2519 and *p* = 0.0447 for DS), respectively (Figure S2A,B and Table S4). In contrast, other hexoses showed weak and non-significant correlations with the GL. Further comparative analysis identified specific vegetables such as raw fava beans, ash guard, cabbage, bitter gourd, Brussels sprout, and watercress that exhibited low levels of both TC and DS. These vegetables demonstrated a stronger association with the GL reduction, clustering closely along the regression line and near the *Y*-axis in both the TC and DS analysis (Figure S2A,B). This graphical distribution indicates a consistent relationship between low carbohydrate content and reduced GL, supporting the hypothesis that incorporating these vegetables into the diet may contribute to improved glycemic regulation. These findings underscore the potential role of low-carbohydrate vegetables in regulating healthy postprandial glucose responses. However, further mechanistic studies are needed to elucidate the biological mechanism.

However, content-to-fiber ratios did not display significant correlation across hexoses or complex carbohydrates in relation to GL. Even the TC-to-DF ratio failed to exhibit a significant positive correlation with the GL, other hexoses also exhibited negative non-significant associations, as reflected in their Pearson’s r and *p* values (Figure S2C,D and Table S5). TS exhibited a negative and non-significant correlation with GL in both independent and when expressed as a ratio to fiber. Notably, neither individual hexoses nor complex carbohydrates showed a significant relationship with GL, suggesting these variables may have limited role on GL determination compared to their more substantial role in affecting the GI.

Further examinations using net carbohydrate content revealed a stronger correlation between individual hexoses and GL, highlighting their potential role in GL determination. However, TC and DS did not show statistically significant correlations with GL, in contrast to their strong positive association with GI. These inverse trends were supported by Pearson’s correlation coefficients r- and *p*-values, as presented in Table S6 (Figure S3A,B). Similarly, net carbohydrate-to-DF ratios failed to demonstrate significant correlations with GL. Interestingly, both TC and DS showed weak, non-significant, and directionally inconsistent correlations with GL (Figure S3C,D and Table S7). Other hexoses, including the simple hexoses such as glucose, fructose, and sucrose, also exhibited linear inverse, non-significant correlations with GL. Data analysis displayed a non-significant Pearson’s r and *p* values summarized in Table S6. Graphical representation showed clustering of TC and DS data points near the x- and y-axes, whereas data points for other sugars were more widely dispersed across both sides of the *x*-axis, indicating weak and inverse associations with GL (graphs are not included). The overall findings suggest an inverse relationship between GL and various vegetable sugar contents. Simple sugars such as glucose, fructose, and sucrose showed a consistent negative correlation with both GI and GL. In contrast, TC and DS were strongly and positively associated with GI but not with GL, indicating that different sugar components of vegetables contribute differently to GR.

**Figure 5:**
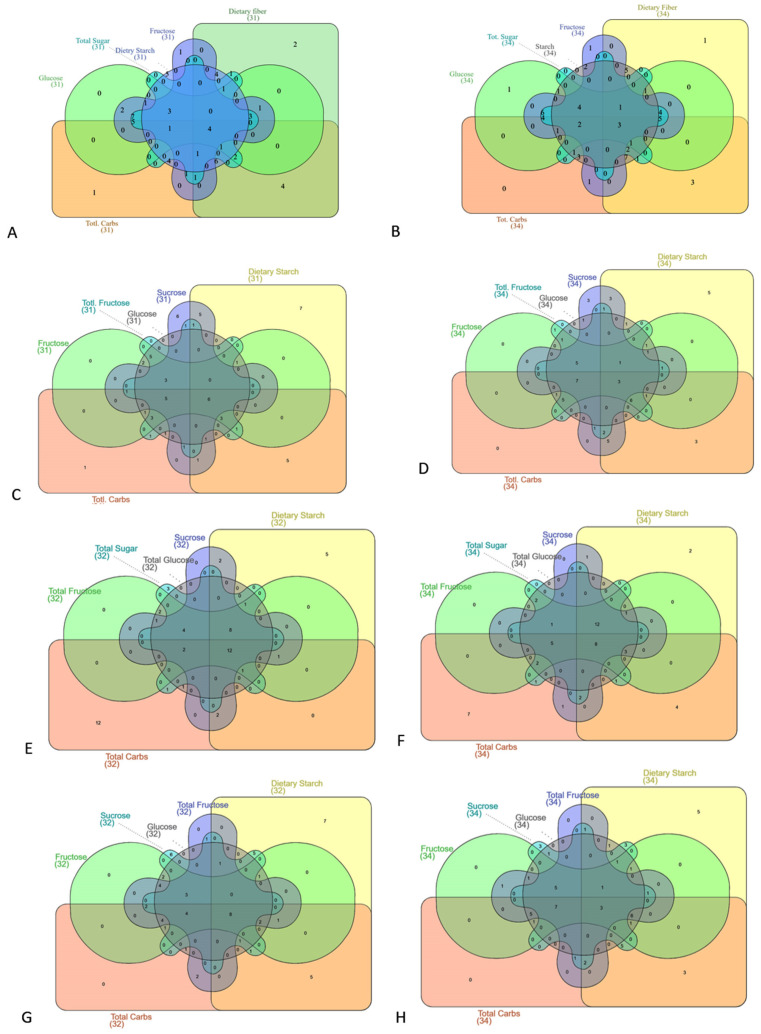
Venn diagrams illustrating the distribution of common vegetables across different carbohydrate categories. (**A**) Total carbohydrates with low sugar content. (**B**) Total carbohydrates with high sugar content. (**C**) Carbohydrate-to-fiber ratios with low sugar content. (**D**) Carbohydrate-to-fiber ratios with high sugar content. (**E**) Available carbohydrates with low sugar content. (**F**) Available carbohydrates with high sugar content. (**G**) Available carbohydrate-to-fiber ratios with low sugar content. (**H**) Available carbohydrate-to-fiber ratios with high sugar content. Numbers indicate vegetable types included in the analysis, mentioned in Table 1.

We analyzed the distribution of common vegetables across various groups based on their minimum and maximum sugar contents to identify those with the lowest and highest carbohydrate levels. Six types of sugars were evaluated across different vegetable categories, and the results were observed using Venn diagrams (Figure 5A–H). In the normal carbohydrate category, rocket (arugula), celery, lettuce, and spinach showed the lowest carbohydrate content. In contrast, green beans, snake beans, and sweet potatoes consistently exhibited the highest values among TC, DF, DS, glucose, fructose, and TS (Figure 5A,B). Similarly, for the carbohydrate-to-fiber ratio, green chili, parsley, kale, rocket, red chili, and mushrooms had the lowest ratios, while spring onions, pumpkins, and cucumbers displayed the highest ratios (Figure 5C,D). Additionally, in the net carbohydrate category, twelve vegetables, including fava beans, watercress, Brussels sprouts, green chili, basil, rocket, kale, bitter gourd, parsley, chicory, string beans, and red potatoes, demonstrated low available carbohydrate content. Conversely, eight vegetables, namely raw fennel, green, orange, red, and yellow bell peppers, pumpkin, white onion, and parsnip, showed the highest carbohydrate content (Figure 5E,F). Finally, in the available carbohydrate-to-fiber category, green and red chili, parsley, kale, rocket, chicory, spinach, and mushrooms had the lowest carbohydrate-to-fiber ratios. In contrast, spring onions, pumpkins, and cucumbers exhibited the highest ratios (Figure 5G,H).

Additionally, several vegetables were identified as statistical outliers due to exceptionally high or low values in TC, DS, glucose, or sucrose content. Specifically, six vegetables were outliers for TC (such as blackeye peas, cassava, garlic, kidney bean, taro root, tapioca), six were as glucose outliers (blackeye peas, kidney bean, green and red chili, potato, and red potato), twelve were as DS outliers (blackeye peas, cassava, garlic, ginger, kidney bean, parsnip, green pea, taro root, tapioca, potato, red potato, sweet potato), and seven were as sucrose outliers (beet root, carrot, kohlrabi, red onion, parsnip, green pea, sweet potato). Despite their outlier status, these vegetables are commonly consumed and widely used in various diets and vegetable-based products. In conclusion, these findings underscore that TC and DS are key determinants of GI in vegetables, while hexoses appear to have minimal influence on both GI and GL. Dietary fiber likely plays a central role in modulating these relationships and may contribute to the complexity of glycemic responses associated with the consumption of vegetables.

## 4. Discussion

Vegetables are staple components of the human diet, providing essential dietary fiber, vitamins, and minerals, and a diverse array of phytochemicals, particularly in raw and leafy vegetables. Compared to “Western” diets, which are often high in sugars, saturated fats, and animal proteins, traditional “Asian” diets are richer in vegetables and legumes. However, a global dietary shift toward “Western” style processed and high-fat foods has been linked to an increased prevalence of non-communicable chronic diseases, including obesity, T2DM, CVD, atherosclerosis, and various cancers. These conditions have been partially associated with gut microbiota dysbiosis (an imbalance in the gut microbiome) [57]. Emerging evidence suggests that fermented foods and plant-based diets may help mitigate the risks associated with “Western” dietary patterns by improving nutritional and functional profiles [58,59]. There are many health organizations, such as the Food and Agriculture Organization (FAO), WHO, USDA, and the European Food and Safety Authority (EFSA), that strongly advocate for the regular consumption of fruits and vegetables due to their well-documented health benefits.

While vegetables are widely consumed and associated with antioxidant and anti-inflammatory effects, optimal intake levels and serving sizes in relation to all-cause and cause-specific mortality remain to be clearly defined. Findings from two large U.S. cohort studies suggest that consuming at least five servings of fruits and vegetables daily (two fruit and three vegetable servings) is associated with reduced mortality risk [22]. However, the impact of specific vegetable types and portion sizes on postprandial glucose responses is less well understood. The nutritional composition of vegetables varies significantly among their different parts, including leaves, shoots, tubers, and bulbs. Notably, leafy vegetables have been shown to elicit significantly lower postprandial glucose area under the curve (AUC) responses compared to other plant parts such as stems and tubers. Therefore, identifying the key nutritional components that modulate the GI of vegetables is critical for dietary strategies aimed at glycemic control and chronic disease prevention.

GI is a key determinant of physiological homeostasis, particularly in regulating blood glucose levels. Research suggests that the proportion of carbohydrates, especially starch and DF, is critical for assessing the quality of food. Diets rich in DF and low in carbohydrates are linked with a reduced risk of chronic diseases [46]. However, the methods used to accurately determine the GI remain uncertain. Conventionally, GI is calculated based on available carbohydrates derived from TC minus DF, and the resulting carbohydrate is compared to the area under the curve (AUC) for an equivalent amount of glucose or white bread as a reference [60]. Numerous studies have shown that low-GI diets improve glucose and lipid metabolism, aid weight control, and reduce obesity risk [61]. Low-GI foods also moderate postprandial blood glucose, lower insulin demand, reduce blood lipids, enhance colonic fermentation, and promote satiety [62]. A recent meta-analysis has shown that an average GI below ≈ 49 and an average dietary GL below ≈ 92 are associated with a lower risk of chronic disease [63].

In this study, we analyzed the vegetable-based carbohydrates, including TC, TS, and DS, and their ratios to DF, comparing these ratios to the net carbohydrate content of vegetables. The findings indicated that the net carbohydrate and their ratios to DF offer a more suitable predictor of GI than net carbohydrates alone, consistent with prior results in fruits and grains [16]. Notably, both TC and the DS, along with their ratios to DF, showed a stronger correlation with GI than hexoses or TS and their ratios to DF. These results highlight the critical role of the DF in modulating GI across plant-based foods. While TC was significantly correlated with GI, the vegetable hexoses also showed inverse associations and clustered along the line of regression, indicating the possibility of additional factors, such as complex carbohydrates, fermentation, polyphenols, and cooking procedures. Recent research highlights the potential of fortifying foods with leafy vegetable powders to enhance fiber, antioxidants, flavonoids, phenolics, and minerals, thereby improving nutritional value and lowering GI. For example, fortifying yellow cassava pasta with amaranth and fluted pumpkin leaf powder increased iron, zinc, phenolics, and flavonoids, contributing to a lower GI [64].

The TC-to-DF ratio showed a strong association with GI, with most vegetables clustering closely along the regression line, reinforcing DF’s importance as a predictor of GI. Similar trends were observed for available carbohydrates, with consistent spatial distributions across vegetables, except for a few, such as fava bean (raw), basil (raw), bitter gourd, and watercress. Both TC-to-DF and net TC-to-DF ratios exhibited comparable trends, reinforcing the central role of DF in modulating GI.

In contrast, TS exhibited a weak, inverse, and non-significant correlation with GI. However, the TS-to-DF ratio improved both correlation strength and regression fit, though the relationship remained negative with scattered data points. Notably, the net DS-to-DF showed a stronger relationship with GI than the net TS-to-DF ratio. However, DS displayed a better correlation with GI compared to TS alone and the ratio with fiber. This relationship further improved when the DS-to-DF ratio was applied, resulting in a tighter clustering of data points along the regression line. Similarly, available (net) starch correlated significantly with GI, and this relationship strengthened when expressed as a DS-to-DF ratio, confirming the moderating influence of DF on starch digestibility and GI.

GL is another key metric associated with the postprandial blood glucose levels. It represents the product of the food’s GI and the amount of available carbohydrate per serving. GL shows stronger associations with macronutrients such as proteins, lipids, fiber, TC, and its individual components. However, the relationship between GI and GL is not always linear. Lower GL diets often achieved by reducing GI, can decrease both fasting blood glucose and glycated proteins in individuals with T2DM [65]. A combination of low GI and GL diet can be achieved by selecting the serving portion, which offers a more practical strategy for managing postprandial glycemia. Several studies have also demonstrated that low GI/GL diets influence post-prandial lipemia, linking them to reduced atherosclerotic risk. In this analysis, many vegetables exhibited a high GI but low GL content. For instance, potato (GI: 86; GL: 10); mushroom (GI: 45; GL: 0.8); parsnip (GI: 85; GL: 15.30); celery (GI: 45; GL: 0.3); and carrot (GI: 47; GL: 3), indicating that foods with a relatively high GI can still have a low GL. Among vegetable components, TC content correlated strongly with both GI and GL, whereas TS failed to reach a significant level. These findings highlight the importance of analyzing the correlation between various sugar contents in vegetable-based foods. These analyses assist in designing diets to optimize glucose regulation, particularly in individuals with metabolic abnormalities.

Vegetables are rich sources of soluble and insoluble DF, both of which play an important role in glycemic regulation. Soluble DF binds to glucose and forms a physical barrier that delays absorption, significantly moderating postprandial blood glucose levels [66,67]. An increased intake of soluble DF has been associated with improved glycemic control in diabetic patients. Animal studies have demonstrated that increased vegetable intake reduces body weight, plasma glucose, and insulin resistance, thereby contributing to improved glucose and insulin homeostasis and potentially preventing diabetes. For instance, broccoli sprout extract, rich in sulforaphane (a glucoraphanin derivative found in cruciferous vegetables), exhibits anti-inflammatory and antioxidant properties, lowering blood glucose and glycated hemoglobin (HbA1c) levels in obese patients with T2DM [68]. Conversely, a high intake of potatoes (GI = 86, GL = 10) has been linked to increased body weight, insulin resistance, and T2DM risk, though these effects vary with cooking and processing methods [69]. Generally, boiled vegetables exhibit higher GI and GL values compared to their fresh counterparts. Potatoes maintain a high GI regardless of the preparation method. Reductions in GI and GL are influenced by the soluble fraction of DF, as thermal processing alters texture through modifications in pectins and alpha-galacto-oligosaccharides in vegetables and legumes. Fresh vegetables typically display lower GI and GL values than boiled or shredded forms, as shredding disrupts cell walls and enhances starch digestibility [70]. Thermal processing, promoting starch gelatinization, improved its digestibility by amylase. Heat treatment further modifies soluble fibers, influencing glycemic response [17]. In contrast, several green leafy vegetables such as agathi, alternanthera, amarantha, basella, cabbage, colocasia, coriander, curry leaves, drumstick, fenugreek, hibiscus, mint, portulaca, rumex, spinach, and tender tamarind leaves show minimal changes in total, insoluble, and soluble DF content after cooking [71]. Thus, the effect of thermal processing on fiber composition may play a key role in modulating the GR of vegetables. Additionally, vegetable juice consumption has been reported to modulate gut microbiota composition. For instance, Bacteroides species associated with weight loss were found to increase following juice intake, accompanied by elevated nitric oxide (NO) levels in blood and urine, potentially improving endothelial function and reducing lipid peroxidation [72].

Notably, brassica vegetables are rich sources of bioactive compounds such as sulforaphane (SFN) and indole-3-carbinol (I3C). This group includes cabbage, broccoli, cauliflower, root vegetables like radish, turnip, and rutabaga, leafy greens such as rocket or kale, as well as condiments like mustard and wasabi. Numerous studies have shown that regular consumption of these vegetables may reduce the risk of several cancers, including breast, prostate, lung, and thyroid cancer. However, findings on their effects, particularly regarding thyroid function following broccoli intake, remain inconsistent. Similarly, evidence concerning other Brassica species such as Brussels sprouts, cauliflower, kohlrabi, rocket, and turnip is inconclusive. Further research is required to clarify potential health risks and benefits, and to establish consumption guidelines based on individual physiological responses [73]. Indole-3-carbinol (I3C) has demonstrated hypoglycemic and antioxidant properties in diabetic models, lowering blood glucose and HbA1c levels, while reducing oxidative stress markers, including thiobarbituric acid reactive substances, lipid hydroperoxides, and conjugated dienes. Concurrently, it enhances antioxidant enzyme activities of superoxide dismutase (SOD), catalase (CAT), and glutathione peroxidase (GSH-Px), and also enhances the levels of vitamins C, E, and glutathione (GSH) [30,69].

Numerous studies have examined the role of DF in glycemic control. However, evidence regarding the digestion and absorption of vegetable carbohydrates remains limited and inconclusive. Increased DF intake has been linked to a 15–30% reduction in all-cause and cardiovascular mortality, as well as lower incidence of coronary heart disease, stroke, and T2DM [74]. Soluble fibers, through their viscosity, delay gastric emptying and glucose absorption, thereby attenuating postprandial glucose excursions [60,67]. This process stimulates glucagon-like peptide-1 (GLP-1) secretion, which promotes glucose-dependent insulin release, inhibits glucagon secretion, enhances β-cell function, improves insulin sensitivity, and prolongs gastric emptying [75,76]. Moreover, fermentation of soluble DF by gut microbiota produces short-chain fatty acids (SCFAs) such as acetate, propionate, and butyrate, which support beneficial microbial growth, exert anti-inflammatory and antioxidant effects, and improve insulin sensitivity [77,78,79]. In contrast, insoluble fibers primarily function as bulking agents, increasing fecal mass and reducing intestinal transit time [80]. Despite these advances, comprehensive data on the interactions among fiber types, starch fractions, and their roles in glucose and carbohydrate metabolism across different vegetables remain scarce.

Several studies have demonstrated the hypoglycemic and antioxidant properties of garlic. Its consumption reduces serum glucose and total cholesterol levels in T2DM db/db mice [81]. Proposed mechanisms include enhanced insulin secretion from β-cells, inhibition of intestinal glucose absorption, increased peripheral glucose utilization, and suppression of fatty acid and cholesterol synthesis [81,82]. Ginger exhibits similar hypoglycemic and hypolipidemic effects, primarily due to its ethanolic constituents that improve insulin sensitivity. It also counteracts serotonin-induced hyperglycemia and hypoinsulinemia by antagonizing serotonin receptors [83]. Onion intake has likewise been associated with reductions in total and LDL cholesterol and improved insulin activity in db/db mice [84]. In our analysis, onion, ginger, and garlic showed strong associations with glycemic index across different carbohydrate profiles. Beyond glycemic regulation, the intake of onions and garlic has been linked to a reduced risk of stomach, colon, ovarian, and breast cancers. Carrot, rich in flavonoids, polyacetylenes, carotenoids, vitamins, and minerals, demonstrates protective effects against prostate cancer [85]. Overall, these findings suggest that incorporating these vegetables into daily diets may improve insulin activity, enhance glycemic control, and confer broader metabolic benefits. However, further evidence is required to substantiate these effects in humans.

Despite the recognized health benefits of vegetable consumption, inconsistencies persist due to variations in study design, population characteristics, and nutrient composition. Low-GI diets may lower diabetes risk by reducing oxidative stress, as reflected by decreased urinary excretion of 8-hydroxy prostaglandin F2α [86]. Elevated reactive oxygen species (ROS) contribute to hyperglycemia-induced complications, including insulin resistance, dyslipidemia, and β-cell dysfunction [87]. A “portfolio diet,” which emphasizes foods known to reduce cardiovascular disease risk, has shown beneficial effects on C-reactive protein (CRP) and LDL cholesterol [88]. Animal studies further suggest that a higher intake of vegetables reduces body weight, plasma glucose, and insulin resistance. The synergistic action of nutrients and phytochemicals, particularly dietary fiber, may underlie these effects [38]. Vegetable-derived fiber delays gastric emptying [89], and glycemic responsiveness [90], and may reduce inflammation [32] and weight gain [33]. Our findings revealed that vegetables such as parsley, kale, rocket, green and red chili, mushrooms, bitter gourd, and chicory are low in total carbohydrates and hexoses, suggesting that their inclusion in daily diets may improve insulin sensitivity and reduce the risk of diabetes. Additionally, fortifying grain- and legume-based foods with these vegetables can enhance insulin secretion and improve GR, thereby mitigating adverse metabolic effects.

## 5. Conclusions

Numerous epidemiological studies have demonstrated that a high intake of vegetable-based foods is associated with a reduced risk of chronic diseases. A meta-analysis indicated that increased vegetable consumption significantly lowers the risk of ischemic stroke and shows a moderate protective effect against T2DM. In contrast, some studies have demonstrated a weaker association between vegetable intake and hemorrhagic stroke and esophageal cancer, even at high levels of vegetable consumption [91]. Our investigation further supports the health-promoting potential of vegetables by demonstrating that TC and DS content play significant roles in determining the GI of various vegetables. Notably, the fiber ratio also exhibited a strong correlation with GI, suggesting that TC and DS, alongside DF, are critical determinants of the glycemic impact of vegetables. Several green leafy vegetables, such as agathi, alternanthera, amaranth, basella, cabbage, colocasia, coriander, curry leaves, drumstick, fenugreek, hibiscus, mint, portulaca, rumex, and spinach, retain their fiber content even after thermal processing. This makes them suitable for maintaining glycemic stability [71]. Additionally, commonly consumed low-carbohydrate vegetables such as celery, spinach, lettuce, rocket, kale, parsley, chilies (green and red), and mushrooms exhibit strong negative associations with GI, making them promising options for reducing postprandial glucose responses.

Our findings suggest that the carbohydrate-to-fiber ratio may serve as a more precise and practical metric for assessing the glycemic impact of vegetables. Additionally, fiber ratio offers a useful framework for dietary planning, particularly for individuals managing diabetes or body weight. It is important to note that dietary interventions are most effective when adopted early in life, with diminishing benefits when initiated later, around the age of eighty [92]. To further validate the practical relevance of our findings, additional investigations in clinical populations are essential. Specifically, studying the effects of daily vegetable intake, such as the 400 g/day recommended by the WHO, and common cooking methods on individual glycemic responses will improve dietary guidance [93]. Our study provides new insights into how the carbohydrate and fiber content of vegetables, along with their preparation methods, affect the glycemic response. By identifying vegetables and cooking techniques that lower GI and GL, we can develop more targeted, evidence-based dietary recommendations that reinforce the health benefits of plant-based diets.

## Figures and Tables

**Table 1:** Carbohydrate content (g) of various vegetables (n = 65) and their ratios to dietary fiber expressed per 100 g per serving, along with the Glycemic Index (GI), Glycemic Load (GL).

S. No.	Vegetables	Carbohydrate Content (g)											Carbohydrate-to-Fiber Ratio (*g*/*g*)							
		Glucose	Total Glucose (TG)	Fructose	Total Fructose (TF)	Sucrose	Total Sugar (TS)	Total carbohydrate (TC)	Starch	Glycemic Index (GI)	Glycemic load (GL)	Dietary Fiber (DF)	Glucose	Total Glucose (TG)	Fructose	Total Fructose (TF)	Total Sugar (TS)	Total Carbohydrate (TC)	Starch	Sucrose
1	Artichoke	0.4	0.500	0.3	0.40	0.2	1.1	11.000	0.4	20	1.2	5.40	0.074	0.093	0.056	0.074	0.204	2.037	0.1	0.0370
2	Ash Gourd	0.900	0.900	1.10	1.10	0.00	2.00	2.100	0.1	15	0.70	0.50	1.800	1.800	2.200	2.200	4.000	4.200	0.2	0.0000
3	Asparagus raw	0.650	0.765	1.00	1.12	0.23	1.88	3.880	0	8	1	2.20	0.295	0.348	0.455	0.507	0.855	1.764	0.0	0.1045
4	Basil raw	0.000	0.000	0.00	0.00	0.00	0.00	3.500	0.2	5.00	0.10	3.30	0.000	0.000	0.000	0.000	0.000	1.061	0.1	0.0000
5	Blackeye peas (Cowpeas)	0.000	0.000	0.00	0.00	0.00	3.00	59.600	37.40	52	13	10.70	0.000	0.000	0.000	0.000	0.280	5.570	3.5	0.0000
6	Beets Raw	0.300	2.445	0.51	2.66	4.29	5.10	8.790	0	30	2.6	3.10	0.097	0.789	0.165	0.856	1.645	2.835	0.0	1.3839
7	Bitter gourd	0.1	0.100	0	0.00	0	0.1	3.700	0.1	18	6.14	3.30	0.030	0.030	0.000	0.000	0.030	1.121	0.0	0.0000
8	Bottle gourd	0.9	0.900	0.8	0.80	0	1.7	3.100	0.3	15	0.02	1.20	0.750	0.750	0.667	0.667	1.417	2.583	0.3	0.0000
9	Broad bean	0.2	0.400	0.1	0.30	0.4	1.5	17.000	1.3	40	7.2	7.10	0.028	0.056	0.014	0.042	0.211	2.394	0.2	0.0563
10	Broccoli	0.580	0.585	0.82	0.83	0.01	1.40	6.270	0	15	0.5	2.40	0.242	0.244	0.342	0.344	0.583	2.613	0.0	0.0042
11	Brussels	0.810	1.040	0.93	1.16	0.46	2.20	9.620	0	15	1.1	4.80	0.169	0.217	0.194	0.242	0.458	2.004	0.0	0.0958
12	Brussels Sprouts	0.81	1.040	0.93	1.16	0.46	3.20	9.62	0	15	0.3	4.7	0.188	0.242	0.216	0.270	0.667	2.004	0.0	0.1070
13	Cabbage	1.700	1.740	1.50	1.54	0.08	3.20	3.510	0	6	7.5	1.30	1.308	1.338	1.154	1.185	2.462	2.700	0.0	0.0615
14	Cabbage green	1.000	1.000	1.30	1.30	0.00	2.30	6.380	0.1	15	0.9	2.55	0.392	0.392	0.510	0.510	0.902	2.502	0.0	0.0000
15	Cabbage Red	1.700	2.000	1.57	1.87	0.60	3.80	6.790	0	32	1	2.60	0.654	0.769	0.604	0.720	1.462	2.612	0.0	0.2308
16	Carrot	1.000	2.350	1.00	2.35	2.70	4.70	9.080	0	47	3	2.90	0.345	0.810	0.345	0.810	1.621	3.131	0.0	0.9310
17	Cassava	0.2	0.600	0.2	0.60	0.8	1.2	30.400	29.2	55	20.9	1.80	0.111	0.333	0.111	0.333	0.667	16.889	16.2	0.4444
18	Cauliflower	1.400	1.400	1.40	1.40	0.00	2.80	4.720	0.5	15	0.8	1.90	0.737	0.737	0.737	0.737	1.474	2.484	0.3	0.0000
19	Celery	0.400	0.440	0.37	0.41	0.08	1.30	3.000	0	45	0.3	1.58	0.253	0.278	0.234	0.259	0.823	1.899	0.0	0.0506
20	Chayote	1.4	1.400	1.7	1.70	0	3.1	3.600	0.5	50	2.3	1.40	1.000	1.000	1.214	1.214	2.214	2.571	0.4	0.0000
21	Chicory	0.3	0.300	0.4	0.40	0	0.7	4.700	0.1	15	0.6	4.00	0.075	0.075	0.100	0.100	0.175	1.175	0.0	0.0000
22	Chili green	0.6	0.650	0.3	0.35	0.1	1	13.090	0	15	124	12.90	0.047	0.050	0.023	0.027	0.078	1.015	0.0	0.0078
23	Chili Red	1.9	1.900	2.3	2.30	0	4.2	14.800	0	15	1.4	10.60	0.179	0.179	0.217	0.217	0.396	1.396	0.0	0.0000
24	Courgette	1.100	1.125	1.40	1.43	0.05	2.50	1.800	0	20	1.7	0.90	1.222	1.250	1.556	1.583	2.778	2.000	0.0	0.0556
25	Cucumber	0.760	0.775	0.87	0.89	0.03	1.70	3.600	0.83	15	0.4	0.50	1.520	1.550	1.740	1.770	3.400	7.200	1.7	0.0600
26	Dill weed	0.8	0.800	0.4	0.40	0	1.2	5.800	1.3	25	1.6	3.30	0.242	0.242	0.121	0.121	0.364	1.758	0.4	0.0000
27	Egg Plant	1.580	1.710	1.54	1.67	0.26	3.53	5.880	0	20	1.7	3.00	0.527	0.570	0.513	0.557	1.177	1.960	0.0	0.0867
28	Fennel raw	1.400	1.650	1.40	1.65	0.50	3.30	5.100	0	15.00	1.10	1.80	0.778	0.917	0.778	0.917	1.833	2.833	0.0	0.2778
29	Fava beans raw	0.2	0.400	0.1	0.30	0.4	9.21	17.60	1.3	40	7.5	7.10	0.028	0.056	0.014	0.042	1.228	2.347	0.2	0.0563
30	Garlic	0.420	0.420	0.62	0.62	0.00	3.70	28.200	35	30	31	2.70	0.156	0.156	0.230	0.230	1.370	10.444	13.0	0.0000
31	Ginger	0.8	0.800	0.9	0.90	0	1.7	7.600	3.1	15	0.6	2.80	0.286	0.286	0.321	0.321	0.607	2.714	1.1	0.0000
32	Green beans	1.330	1.370	0.93	0.97	0.08	3.00	7.060	0.7	20	1	3.53	0.377	0.388	0.263	0.275	0.850	2.000	0.2	0.0227
33	Kale	0.400	0.400	0.40	0.40	0.00	0.80	4.420	0	15.00	3.00	4.10	0.098	0.098	0.098	0.098	0.195	1.078	0.0	0.0000
34	Kidney Beans (Red)	0.3	0.700	0.1	0.50	0.8	1.2	23.800	13	35	18.4	9.10	0.033	0.077	0.011	0.055	0.132	2.615	1.4	0.0879
35	Kohlrabi	1.8	2.250	1.5	1.95	0.9	4.2	4.200	0	20	2.1	3.30	0.545	0.682	0.455	0.591	1.273	1.273	0.0	0.2727
36	Leek	1.5	1.850	1.5	1.85	0.7	3.7	6.800	0	15	1.2	3.10	0.484	0.597	0.484	0.597	1.194	2.194	0.0	0.2258
37	Lettuce	0.390	0.390	0.80	0.80	0.00	1.19	3.240	0	15	0.5	1.80	0.217	0.217	0.444	0.444	0.661	1.800	0.0	0.0000
38	Lima Bean	0.4	0.600	1	1.20	0.4	1.8	2.300	0.3	32	8	2.30	0.174	0.261	0.435	0.522	0.783	1.000	0.1	0.1739
39	Mushrooms beech	0.220	0.220	0.14	0.14	0.00	0.36	6.760	0	45	0	3.10	0.071	0.071	0.046	0.046	0.117	2.181	0.0	0.0000
40	Okra	0.320	0.620	0.57	0.87	0.60	1.50	7.500	0.34	20	1	3.20	0.100	0.194	0.178	0.272	0.469	2.344	0.1	0.1875
41	Onion Spring	1.900	2.050	2.30	2.45	0.30	4.50	7.300	0.1	15	1.6	2.60	0.731	0.788	0.885	0.942	1.731	2.808	0.0	0.1154
42	Onion red	2.290	3.130	1.79	2.63	1.68	5.67	9.930	0	15	1.6	3.97	0.577	0.788	0.451	0.662	1.428	2.501	0.0	0.4232
43	Onion white	2.630	2.925	2.52	2.82	0.59	5.76	7.680	0	15	1.6	1.20	2.192	2.438	2.100	2.346	4.800	6.400	0.0	0.4917
44	Parsnip	0.800	2.400	0.80	2.40	3.20	4.80	13.600	5.2	85.00	15.30	3.60	0.222	0.667	0.222	0.667	1.333	3.778	1.4	0.8889
45	Parsley	0.100	0.150	0.20	0.25	0.10	0.40	6.200	0	15.00	0.90	5.80	0.017	0.026	0.034	0.043	0.069	1.069	0.0	0.0172
46	Pea green	0.120	2.620	0.39	2.89	5.00	5.70	12.700	4.48	15	1.5	5.97	0.020	0.439	0.065	0.484	0.955	2.127	0.8	0.8375
47	Pepper bell green	1.100	1.100	1.10	1.10	0.00	2.20	4.780	0	15	1	0.90	1.222	1.222	1.222	1.222	2.444	5.311	0.0	0.0000
48	Pepper bell orange	1.900	1.900	2.30	2.30	0.00	4.20	6.000	0	15	1	1.00	1.900	1.900	2.300	2.300	4.200	6.000	0.0	0.0000
49	Pepper bell red	1.900	1.900	2.30	2.30	0.00	5.30	6.650	0	15	1	1.20	1.583	1.583	1.917	1.917	4.417	5.542	0.0	0.0000
50	Pepper bell Yellow	1.900	1.900	2.30	2.30	0.00	4.20	6.600	0	15	1	1.10	1.727	1.727	2.091	2.091	3.818	6.000	0.0	0.0000
51	Potato	0.210	0.275	0.31	0.38	0.13	0.65	14.700	11.9	86	10	13.80	0.015	0.020	0.022	0.027	0.047	1.065	0.9	0.0094
52	Potato red	0.180	0.310	0.22	0.35	0.26	0.66	16.300	9	82	29	13.80	0.013	0.022	0.016	0.025	0.048	1.181	0.7	0.0188
53	Pumpkin	1.200	1.350	1.10	1.25	0.30	2.60	5.100	1.4	52	4	1.10	1.091	1.227	1.000	1.136	2.364	4.636	1.3	0.2727
54	Radish	1.100	1.100	0.80	0.80	0.00	1.90	3.000	0	15	0.5	1.10	1.000	1.000	0.727	0.727	1.727	2.727	0.0	0.0000
55	Rocket (arugula)	0.300	0.300	0.00	0.00	0.00	0.30	2.700	0	15	0.3	2.40	0.125	0.125	0.000	0.000	0.125	1.125	0.0	0.0000
56	Snake beans	1.1	1.250	1.2	1.35	0.3	2.60	8.400	0.7	43	1	3.60	0.306	0.347	0.333	0.375	0.722	2.333	0.2	0.0833
57	String bean	1.1	1.250	1.2	1.35	0.3	2.6	5.300	0.7	30	1.1	3.70	0.297	0.338	0.324	0.365	0.703	1.432	0.2	0.0811
58	Spinach mature	0.110	0.145	0.15	0.19	0.07	0.42	3.630	0	15	0.3	2.20	0.050	0.066	0.068	0.084	0.191	1.650	0.0	0.0318
59	Shallot	1.500	1.500	1.60	1.60	0.00	3.10	6.000	0	15	2.5	2.90	0.517	0.517	0.552	0.552	1.069	2.069	0.0	0.0000
60	Sweet potato	0.980	2.510	0.93	2.46	3.06	4.97	17.300	8.5	61	17	4.44	0.221	0.565	0.209	0.554	1.119	3.896	1.9	0.6892
61	Taro root	0.2	0.550	0.2	0.55	0.7	1.10	23.400	22.3	48	12.7	3.50	0.057	0.157	0.057	0.157	0.314	6.686	6.4	0.2000
62	Tapioca	0	0.000	0	0.00	0	0.00	88.700	79.3	78	20	0.90	0.000	0.000	0.000	0.000	0.000	98.556	88.1	0.0000
63	Turnip raw	0.520	0.520	0.69	0.69	0.00	1.21	6.430	0.2	30	1.9	1.80	0.289	0.289	0.383	0.383	0.672	3.572	0.1	0.0000
64	Water Cress	0.4	0.500	0.1	0.20	0.2	4.35	5.530	0.1	15	0.1	1.10	0.105	0.132	0.026	0.053	3.955	5.027	0.0	0.0526
65	Zucchini	1.070	1.095	1.38	1.41	0.05	2.50	4.200	0	15	0.5	1.10	0.973	0.995	1.255	1.277	2.273	3.818	0.0	0.0455

**Note:** Total glucose content was calculated as the sum of free glucose and half of the sucrose content. Similarly, total fructose content was determined as the sum of free fructose and one-half of the sucrose content. Finally, total sugar was calculated as the sum of glucose, fructose, and sucrose. The data presented are mean values derived from published literature or a single data point obtained from reliable food composition databases showed in method section.

**Table 2:** PCA of various components in vegetables (n = 65).

PC Summary	PC1	PC2	PC3	PC4	PC5	PC6	PC7	PC8
Eigenvalue	4.774	1.944	1.279	0.8096	0.09674	0.06607	0.03112	2.055 × 10^−6^
Proportion of variance	53.04%	21.60%	14.21%	9.00%	1.07%	0.73%	0.35%	2.28 × 10^−5^%
Cumulative proportion of variance	53.04%	74.64%	88.85%	97.85%	98.92%	99.65%	100.00%	100.00%
Components Selection	Selected	Selected						

**Table 3:** MLRA assessment for the correlation of carbohydrate content and the carbohydrate-to-dietary fiber ratio with GI in various vegetables (n = 65).

Values	Carbohydrate Content (MLRA)									Carbohydrate-to-Fiber Ratio (MLRA)							
	Glucose	Total Glucose	Fructose	Total Fructose	Total Sugar	Total Carbohydrates	Dietary Fiber	Starch	Sucrose	Glucose	Total Glucose	Fructose	Total Fructose	Total Sugar	Total Carbohydrates	Starch	Sucrose
R	−0.3196	−0.1097	−0.3240	−0.1252	−0.0868	0.5307	0.3649	0.5276	0.2384	−0.3063	−0.2411	−0.3012	−0.2403	−0.2403	0.3543	0.3869	0.2541
R^2^	0.1022	0.01204	0.1049	0.01568	0.00754	0.2817	0.1332	0.2783	0.0568	0.0937	0.0581	0.0907	0.0599	0.0577	0.1256	0.1497	0.0645
*p*-values	0.0094	0.3842	0.0085	0.3203	0.4914	<0.0001	0.0028	<0.0001	0.0559	0.0131	0.0530	0.0148	0.0493	0.0538	0.0038	0.0015	0.0411
Significance	**	ns	**	ns	ns	****	**	****	ns	*	ns	*	*	ns	**	**	*

Note: Asterisks (*) indicate levels of statistical significance “****” *p* < 0.0001 (highly significant), “**” *p* < 0.01 (moderately significant), “*” *p* < 0.05 (low significance), ns = not significant.

**Table 4:** MLRA assessment of GI in various vegetables (n = 65) based on individual carbohydrate content, and their ratio to dietary fiber.

Values	Net Carbohydrate Content (MLRA)								Net Carbohydrate-to-Fiber Ratio (MLRA)							
	Glucose	Total Glucose	Fructose	Total Fructose	Total Sugar	Total Carbohydrate	Starch	Sucrose	Glucose	Total Glucose	Fructose	Total Fructose	Total Sugar	Total Carbohydrate	Starch	Sucrose
R	−0.3984	−0.3631	−0.3984	−0.3636	−0.3415	0.4612	0.4358	−0.2798	−0.3063	−0.2411	−0.3012	−0.2449	−0.2403	0.3543	0.3868	0.2539
R^2^	0.1587	0.1319	0.1587	0.1322	0.1166	0.2127	0.1900	0.07831	0.09379	0.05814	0.09070	0.05998	0.05775	0.1256	0.1496	0.06449
*p*-values	0.0010	0.0029	0.0010	0.0029	0.0054	0.0001	0.0003	0.0240	0.0131	0.0530	0.0148	0.0493	0.0538	0.0038	0.0015	0.0412
Significance	**	**	**	**	**	***	***	*	*	ns	*	*	ns	**	**	*

Note: Asterisks (*) indicate levels of statistical significance “***” *p* < 0.001 (highly significant), “**” *p* < 0.01 (moderately significant), “*” *p* < 0.05 (low significance), ns = not significant.

**Table 5:** Statistical analysis of carbohydrate content and GI in various vegetables (n = 65).

Values	Glucose	Total Glucose	Fructose	Total Fructose	Total Sugar	Total Carbohydrates	Dietary Starch	Sucrose	Dietary Fiber
R	−0.3196	−0.1097	−0.3240	−0.1252	−0.08687	0.5367	0.5238	0.2384	0.3646
R^2^	0.1022	0.01204	0.1049	0.01568	0.007547	0.2881	0.2791	0.05682	0.1332
*p*-values	0.0094	0.3842	0.0085	0.3203	0.4914	<0.0001	<0.0001	0.0559	0.0028

**Table 6:** Statistical analysis of individual carbohydrate-to-DF ratio and GI in various vegetables (n = 65).

Values	Glucose	Total Glucose	Fructose	Total Fructose	Total Sugar	Total Carbohydrates	Dietary Starch	Sucrose
R	−0.4163	−0.2411	−0.3012	−0.2449	−0.2403	0.3517	0.3869	0.2541
R^2^	0.1733	0.05814	0.09070	0.05998	0.05775	0.1237	0.1497	0.06458
*p*-values	0.0143	0.0530	0.0148	0.0493	0.0538	0.0041	0.0015	0.0411

**Table 7:** Statistical analysis of available carbohydrate content in various vegetables (n = 65).

Values	Glucose	Total Glucose	Fructose	Total Fructose	Total Sugar	Total Carbohydrates	Dietary Starch	Sucrose
R	−0.3984	−0.3631	−0.3984	−0.3636	−0.3415	0.4682	0.4358	−0.2798
R^2^	0.1587	0.1319	0.1587	0.1322	0.1166	0.2192	0.1900	0.07831
*p*-values	0.0010	0.0029	0.0010	0.0029	0.0054	<0.0001	0.0003	0.0240

**Table 8:** Statistical analysis of available carbohydrate content-to-fiber ratio and GI in various vegetables (n = 65).

Values	Glucose	Total Glucose	Fructose	Total Fructose	Total Sugar	Total Carbohydrates	Dietary Starch	Sucrose
R	−0.3063	−0.2411	−0.3012	−0.2449	−0.2403	0.3517	0.3868	0.2539
R^2^	0.09379	0.05814	0.09070	0.05998	0.05775	0.1237	0.1496	0.06449
*p*-values	0.0131	0.0530	0.0148	0.0493	0.0538	0.0041	0.0015	0.0412

## Data Availability

The original contributions presented in the study are included in the article/Supplementary Materials; further inquiries can be directed to the corresponding authors.

## Supplementary Materials

The following supporting information can be downloaded at: https://www.mdpi.com/article/10.3390/foods14213703/s1, Figure S1: MLRA estimation of carbohydrate components in various vegetables (n = 65) in relation to GL; Figure S2: The correlation plot illustrating the relationship between the GL and the carbohydrates content as well as carbohydrate-to-DF ratios in vegetables (n = 65); Figure S3: The correlation plot illustrating the relationship between the GL and vegetable derive available carbohydrates content as well as available carbohydrate-to-DF ratios in vegetables (n = 65); Table S1: Available carbohydrate content of various vegetables, estimated by conventional method; Table S2: MLRA of GL in various vegetables (n = 65) based on carbohydrate content and the carbohydrate-to-dietary fiber ratio; Table S3: MLRA of GL in various vegetables (n = 65) based on individual carbohydrate content excluding fiber (i.e., net carbohydrates), and the ratio of net carbohydrate content to dietary fiber; Table S4: Statistical analysis of carbohydrate content and GL in various Vegetables (n = 65); Table S5: Statistical analysis of individual carbohydrates-to-dietary fiber ratio and GL in various vegetables (n = 65); Table S6: Statistical analysis of individual net available carbohydrate and GL in various vegetables (n = 65); Table S7: Statistical analysis of available carbohydrate content-to-fiber ratio and GL in various vegetables (n = 65).

## Abbreviations

The following abbreviations are used in this manuscript:

AUC	Area under the curve
CRP	C-reactive Protein
CVD	Cardiovascular disease
CQI	carbohydrate quality index
DS	Dietary Starch
DF	Dietary fiber
DRI	Dietary Reference Intake
FAO	Food and Agriculture Organization
FGM/isCGM	Flash/Intermittently scanned continuous glucose monitoring
g	Gram
GI	Glycemic index
GL	Glycemic load
GR	Glycemic response
GLP-1	Glucagon-like peptide
G6P	Glucose-6-phosphatase
HbA1c	Hemoglobin A1c
I3C	Indile-3-carbinol
IDF	Insoluble dietary fiber
KFCD	Korean Food Composition Database
LDL	Low-density lipoprotein
MLRA	Multiple linear regression analysis
PCA	Principal component analysis
PEPCK	Phosphoenolpyruvate carboxykinase
SCFAs	Short-chain fatty acids
SDF	Soluble dietary fiber
SFN	Sulforaphane
T2DM	Type 2 Diabetes mellitus
TC	Total Carbohydrates
TG	Total Glucose
TF	Total Fructose
TS	Total Starch
USDA	U.S. Department of Agriculture
WHO	World Health Organization
