# Supplementary material for: Vegetables and Glycemic Index: Exploring Their Correlation and Health Implications
Source: Foods. 2025 Oct 29;14(21):3703. doi: 10.3390/foods14213703 (PMC12607511; doi:10.3390/foods14213703)
Supplement: Supplementary file 1 [file foods-14-03703-s001.zip › foods-3920701-supplementary.pdf]

Table S1. Available carbohydrate content of various vegetables, estimated by conventional method.

S. No.	Vegetables	Net available carbohydrates (g)								Net Carbohydrates-to-fiber ratio							
		Glucose	Total Glucose (TG)	Fructose	Total Fructose (TF)	Total Sugar (TS)	Total Carbohydrate (TC)	Starch	Sucrose	Glucose	Total Glucose (TG)	Fructose	Total Fructose (TF)	Total Sugar (TS)	Total Carbohydrate (TC)	Starch	Sucrose
1	Artichoke	-5.00	-4.90	-5.10	-5.00	-4.300	5.600	-5.0	-5.20	-0.926	-0.907	-0.944	-0.926	-0.796	1.037	-0.926	-0.963
2	Ash Gourd	0.40	0.40	0.60	0.60	1.500	1.600	-0.4	-0.50	0.800	0.800	1.200	1.200	3.000	3.200	-0.800	-1.000
3	Asparagus raw	-1.55	-1.44	-1.20	-1.09	-0.320	1.680	-2.2	-1.97	-0.705	-0.652	-0.545	-0.493	-0.145	0.764	-1.000	-0.895
4	Basil raw	-3.30	-3.30	-3.30	-3.30	-3.300	0.200	-3.1	-3.30	-1.000	-1.000	-1.000	-1.000	-1.000	0.061	-0.939	-1.000
5	Blackeye peas (Cowpeas)	-10.70	-10.70	-10.70	-10.70	-7.700	48.900	26.7	-10.70	-1.000	-1.000	-1.000	-1.000	-0.720	4.570	2.495	-1.000
6	Beets Raw	-2.80	-0.66	-2.59	-0.45	2.000	5.690	-3.1	1.19	-0.903	-0.211	-0.835	-0.144	0.645	1.835	-1.000	0.384
7	Bitter gourd	-3.20	-3.20	-3.30	-3.30	-3.200	0.400	-3.2	-3.30	-0.970	-0.970	-1.000	-1.000	-0.970	0.121	-0.970	-1.000
8	Bottle gourd	-0.30	-0.30	-0.40	-0.40	0.500	1.900	-0.9	-1.20	-0.250	-0.250	-0.333	-0.333	0.417	1.583	-0.750	-1.000
9	Broad bean	-6.90	-6.70	-7.00	-6.80	-5.600	9.900	-5.8	-6.70	-0.972	-0.944	-0.986	-0.958	-0.789	1.394	-0.817	-0.944
10	Broccoli	-1.82	-1.82	-1.58	-1.58	-1.000	3.870	-2.4	-2.39	-0.758	-0.756	-0.658	-0.656	-0.417	1.613	-1.000	-0.996
11	Brussels	-3.99	-3.76	-3.87	-3.64	-2.600	4.820	-4.8	-4.34	-0.831	-0.783	-0.806	-0.758	-0.542	1.004	-1.000	-0.904
12	Brussels Sprouts	-3.49	-3.26	-3.37	-3.14	-1.100	-2.000	-4.3	-3.84	-0.812	-0.758	-0.784	-0.730	-0.256	-0.465	-1.000	-0.893
13	Cabbage	0.40	0.44	0.20	0.24	1.900	2.210	-1.3	-1.22	0.308	0.338	0.154	0.185	1.462	1.700	-1.000	-0.938
14	Cabbage green	-1.55	-1.55	-1.25	-1.25	-0.250	3.830	-2.5	-2.55	-0.608	-0.608	-0.490	-0.490	-0.098	1.502	-0.961	-1.000
15	Cabbage Red	-0.90	-0.60	-1.03	-0.73	1.200	4.190	-2.6	-2.00	-0.346	-0.231	-0.396	-0.280	0.462	1.612	-1.000	-0.769

16	Carrot	-1.90	-0.55	-1.90	-0.55	1.800	6.180	-2.9	-0.20	-0.655	-0.190	-0.655	-0.190	0.621	2.131	-1.000	-0.069
17	Cassava	-1.60	-1.20	-1.60	-1.20	-0.600	28.600	27.4	-1.00	-0.889	-0.667	-0.889	-0.667	-0.333	15.889	15.222	-0.556
18	Cauliflower	-0.50	-0.50	-0.50	-0.50	0.900	2.820	-1.4	-1.90	-0.263	-0.263	-0.263	-0.263	0.474	1.484	-0.737	-1.000
19	Celery	-1.18	-1.14	-1.21	-1.17	-0.280	1.420	-1.6	-1.50	-0.747	-0.722	-0.766	-0.741	-0.177	0.899	-1.000	-0.949
20	Chayote	0.00	0.00	0.30	0.30	1.700	2.200	-0.9	-1.40	0.000	0.000	0.214	0.214	1.214	1.571	-0.643	-1.000
21	Chicory	-3.70	-3.70	-3.60	-3.60	-3.300	0.700	-3.9	-4.00	-0.925	-0.925	-0.900	-0.900	-0.825	0.175	-0.975	-1.000
22	Chili green	-12.30	-12.25	-12.60	-12.55	-11.900	0.190	-12.9	-12.80	-0.953	-0.950	-0.977	-0.973	-0.922	0.015	-1.000	-0.992
23	Chili Red	-8.70	-8.70	-8.30	-8.30	-6.400	4.200	-10.6	-10.60	-0.821	-0.821	-0.783	-0.783	-0.604	0.396	-1.000	-1.000
24	Courgette	0.20	0.23	0.50	0.53	1.600	0.900	-0.9	-0.85	0.222	0.250	0.556	0.583	1.778	1.000	-1.000	-0.944
25	Cucumber	0.26	0.28	0.37	0.39	1.200	3.100	0.3	-0.47	0.520	0.550	0.740	0.770	2.400	6.200	0.660	-0.940
26	Dill weed	-2.50	-2.50	-2.90	-2.90	-2.100	2.500	-2.0	-3.30	-0.758	-0.758	-0.879	-0.879	-0.636	0.758	-0.606	-1.000
27	Egg Plant	-1.42	-1.29	-1.46	-1.33	0.530	2.880	-3.0	-2.74	-0.473	-0.430	-0.487	-0.443	0.177	0.960	-1.000	-0.913
28	Fennel raw	-0.40	-0.15	-0.40	-0.15	1.500	3.300	-1.8	-1.30	-0.222	-0.083	-0.222	-0.083	0.833	1.833	-1.000	-0.722
29	Fava beans raw	-6.90	-6.70	-7.00	-6.80	-6.400	-4.900	-5.8	-6.70	-0.972	-0.944	-0.986	-0.958	-0.901	-0.690	-0.817	-0.944
30	Garlic	-2.28	-2.28	-2.08	-2.08	1.000	25.500	32.3	-2.70	-0.844	-0.844	-0.770	-0.770	0.370	9.444	11.963	-1.000
31	Ginger	-2.00	-2.00	-1.90	-1.90	-1.100	4.800	0.3	-2.80	-0.714	-0.714	-0.679	-0.679	-0.393	1.714	0.107	-1.000
32	Green beans	-2.20	-2.16	-2.60	-2.56	-0.530	3.530	-2.8	-3.45	-0.623	-0.612	-0.737	-0.725	-0.150	1.000	-0.802	-0.977
33	Kale	-3.70	-3.70	-3.70	-3.70	-3.300	0.320	-4.1	-4.10	-0.902	-0.902	-0.902	-0.902	-0.805	0.078	-1.000	-1.000
34	Kidney Beans (Red)	-8.80	-8.40	-9.00	-8.60	-7.900	14.700	3.9	-8.30	-0.967	-0.923	-0.989	-0.945	-0.868	1.615	0.429	-0.912
35	Kohlrabi	-1.50	-1.05	-1.80	-1.35	0.900	0.900	-3.3	-2.40	-0.455	-0.318	-0.545	-0.409	0.273	0.273	-1.000	-0.727
36	Leek	-1.60	-1.25	-1.60	-1.25	0.600	3.700	-3.1	-2.40	-0.516	-0.403	-0.516	-0.403	0.194	1.194	-1.000	-0.774
37	Lettuce	-1.41	-1.41	-1.00	-1.00	-0.610	1.440	-1.8	-1.80	-0.783	-0.783	-0.556	-0.556	-0.339	0.800	-1.000	-1.000
38	Lima Bean	-1.90	-1.70	-1.30	-1.10	-0.500	0.000	-2.0	-1.90	-0.826	-0.739	-0.565	-0.478	-0.217	0.000	-0.870	-0.826
39	Mushrooms beech	-2.88	-2.88	-2.96	-2.96	-2.738	3.660	-3.1	-3.10	-0.929	-0.929	-0.954	-0.954	-0.883	1.181	-1.000	-1.000
40	Okra	-2.88	-2.58	-2.63	-2.33	-1.700	4.300	-2.9	-2.60	-0.900	-0.806	-0.822	-0.728	-0.531	1.344	-0.894	-0.813

41	Onion Spring	-0.70	-0.55	-0.30	-0.15	1.900	4.700	-2.5	-2.30	-0.269	-0.212	-0.115	-0.058	0.731	1.808	-0.962	-0.885
42	Onion red	-1.68	-0.84	-2.18	-1.34	1.700	5.960	-4.0	-2.29	-0.423	-0.212	-0.549	-0.338	0.428	1.501	-1.000	-0.577
43	Onion white	1.43	1.73	1.32	1.62	4.560	6.480	-1.2	-0.61	1.192	1.438	1.100	1.346	3.800	5.400	-1.000	-0.508
44	Parsnip	-2.80	-1.20	-2.80	-1.20	1.200	10.000	1.6	-0.40	-0.778	-0.333	-0.778	-0.333	0.333	2.778	0.444	-0.111
45	Parsley	-5.70	-5.65	-5.60	-5.55	-5.400	0.400	-5.8	-5.70	-0.983	-0.974	-0.966	-0.957	-0.931	0.069	-1.000	-0.983
46	Pea green	-5.85	-3.35	-5.58	-3.08	-0.270	6.730	-1.5	-0.97	-0.980	-0.561	-0.935	-0.516	-0.045	1.127	-0.250	-0.162
47	Pepper bell green	0.20	0.20	0.20	0.20	1.300	3.880	-0.9	-0.90	0.222	0.222	0.222	0.222	1.444	4.311	-1.000	-1.000
48	Pepper bell orange	0.90	0.90	1.30	1.30	3.200	5.000	-1.0	-1.00	0.900	0.900	1.300	1.300	3.200	5.000	-1.000	-1.000
49	Pepper bell red	0.70	0.70	1.10	1.10	4.100	5.450	-1.2	-1.20	0.583	0.583	0.917	0.917	3.417	4.542	-1.000	-1.000
50	Pepper bell Yellow	0.80	0.80	1.20	1.20	3.100	5.500	-1.1	-1.10	0.727	0.727	1.091	1.091	2.818	5.000	-1.000	-1.000
51	Potato	-13.59	-13.53	-13.49	-13.43	-13.150	0.900	-1.9	-13.67	-0.985	-0.980	-0.978	-0.973	-0.953	0.065	-0.138	-0.991
52	Potato red	-13.62	-13.49	-13.58	-13.45	-13.140	2.500	-4.8	-13.54	-0.987	-0.978	-0.984	-0.975	-0.952	0.181	-0.348	-0.981
53	Pumpkin	0.10	0.25	0.00	0.15	1.500	4.000	0.3	-0.80	0.091	0.227	0.000	0.136	1.364	3.636	0.273	-0.727
54	Radish	0.00	0.00	-0.30	-0.30	0.800	1.900	-1.1	-1.10	0.000	0.000	-0.273	-0.273	0.727	1.727	-1.000	-1.000
55	Rocket (arugula)	-2.10	-2.10	-2.40	-2.40	-2.100	0.300	-2.4	-2.40	-0.875	-0.875	-1.000	-1.000	-0.875	0.125	-1.000	-1.000
56	Snake beans	-2.50	-2.35	-2.40	-2.25	-1.000	4.800	-2.9	-3.30	-0.694	-0.653	-0.667	-0.625	-0.278	1.333	-0.806	-0.917
57	String bean	-2.60	-2.45	-2.50	-2.35	-1.100	1.600	-3.0	-3.40	-0.703	-0.662	-0.676	-0.635	-0.297	0.432	-0.811	-0.919
58	Spinach mature	-2.09	-2.06	-2.05	-2.02	-1.780	1.430	-2.2	-2.13	-0.950	-0.934	-0.932	-0.916	-0.809	0.650	-1.000	-0.968
59	Shallot	-1.40	-1.40	-1.30	-1.30	0.200	3.100	-2.9	-2.90	-0.483	-0.483	-0.448	-0.448	0.069	1.069	-1.000	-1.000
60	Sweet potato	-3.46	-1.93	-3.51	-1.98	0.530	12.860	4.1	-1.38	-0.779	-0.435	-0.791	-0.446	0.119	2.896	0.914	-0.311
61	Taro root	-3.30	-2.95	-3.30	-2.95	-2.400	19.900	18.8	-2.80	-0.943	-0.843	-0.943	-0.843	-0.686	5.686	5.371	-0.800

62	Tapioca	-0.90	-0.90	-0.90	-0.90	-0.900	87.800	78.4	-0.90	-1.000	-1.000	-1.000	-1.000	-1.000	97.556	87.111	-1.000
63	Turnip raw	-1.28	-1.28	-1.11	-1.11	-0.590	4.630	-1.6	-1.80	-0.711	-0.711	-0.617	-0.617	-0.328	2.572	-0.889	-1.000
64	Water Cress	-3.40	-3.30	-3.70	-3.60	-3.100	-3.000	-3.7	-3.60	-0.895	-0.868	-0.974	-0.947	-0.816	-0.789	-0.974	-0.947
65	Zucchini	-0.03	-0.01	0.28	0.31	1.400	3.100	-1.1	-1.05	-0.027	-0.005	0.255	0.277	1.273	2.818	-1.000	-0.955

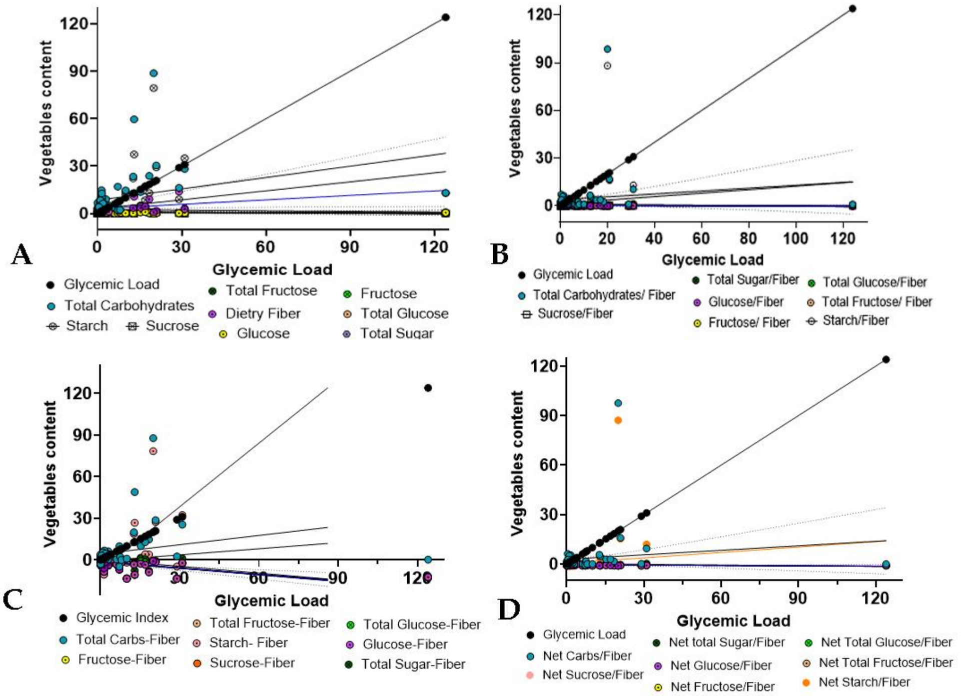

Figure S1. A-D: Multiple linear regression analysis (MLRA) of carbohydrate components in vegetables (n = 65) in relation to glycemic load (GL). (A) MLRA between total carbohydrate contents and GL. (B) MLRA between carbohydrate-to-fiber ratios and GL. (C) Correlation between available carbohydrate contents and GL. (D) Correlation between available carbohydrate-to-fiber ratios and GL. Each data point represents an individual vegetable, with carbohydrate content values on the y-axis and GL on the x-axis.

Table S2. Multiple linear regression analysis of glycemic load (GL) in various vegetables (n = 65) based on carbohydrate content and the carbohydrate-to-dietary fiber ratio.

Values	Carbohydrate Content (MLRA)									Carbohydrate-to-fiber ratio (MLRA)							
	Glucose	Total Glucose	Fructose	Total Fructose	Total Sugar	Total Carbohydrates	Dietary Fiber	Starch	Sucrose	Glucose	Total Glucose	Fructose	Total Fructose	Total Sugar	Total Carbohydrates	Starch	Sucrose
R	-0.1859	-0.1414	-0.2402	-0.1905	-0.1288	0.2937	0.5017	0.2519	0.0152	-0.2152	-0.2144	-0.2134	-0.2140	-0.1981	0.1229	0.1604	-0.0069
R^2	0.0345	0.0199	0.0576	0.0363	0.0166	0.0862	0.2517	0.0634	0.0002	0.0463	0.0459	0.0455	0.0458	0.0392	0.0151	0.0257	4.797e-005
p-values	0.1382	0.2613	0.0540	0.1285	0.3064	0.0176	<0.0001	0.0447	0.9038	0.0852	0.0863	0.0879	0.0869	0.1136	0.3294	0.2017	0.9563
Significance	ns	ns	ns	ns	ns	*	****	*	ns	ns	ns	ns	ns	ns	ns	ns	ns

Table S3. Multiple linear regression analysis of glycemic load (GL) in various vegetables (n = 65) based on individual carbohydrate content excluding fiber (i.e., net carbohydrates), and the ratio of net carbohydrate content to dietary fiber.

Values	Net Carbohydrate Content (MLRA)								Net Carbohydrate-to-fiber ratio (MLRA)							
	Glucose	Total Glucose	Fructose	Total Fructose	Total Sugar	Total Carbohydrates	Starch	Sucrose	Glucose	Total Glucose	Fructose	Total Fructose	Total Sugar	Total Carbohydrates	Starch	Sucrose
R	-0.4985	-0.4969	-0.5058	-0.5043	-0.4736	0.1840	0.1276	-0.4840	-0.2152	-0.2144	-0.2134	-0.2140	-0.1981	0.1229	0.1604	-0.0068
R^2	0.2485	0.2469	0.2558	0.2543	0.2243	0.03387	0.01629	0.2343	0.04630	0.04598	0.04553	0.04580	0.03925	0.01510	0.02573	4.746e-005
p-values	<0.0001	<0.0001	<0.0001	<0.0001	<0.0001	0.1423	0.3110	<0.0001	0.0852	0.0863	0.0879	0.0869	0.1136	0.3294	0.2018	0.9566
Significance	****	****	****	****	****	ns	ns	****	ns	ns	ns	ns	ns	ns	ns	ns

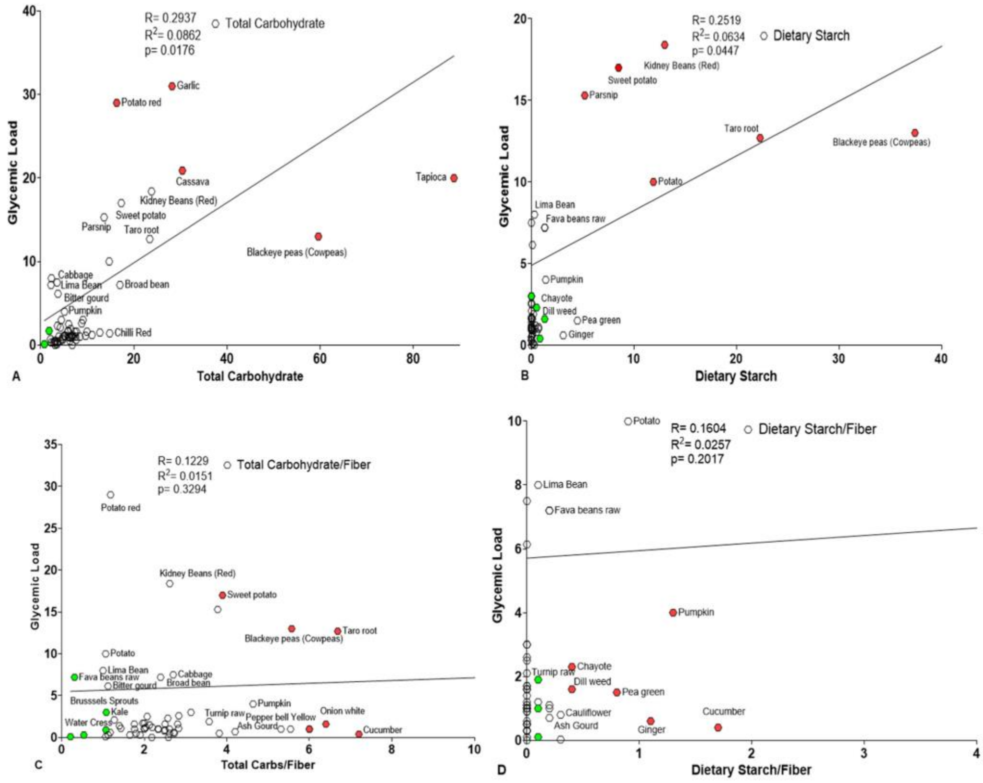

Figure S2. A-D: The correlation plot illustrating the relationship between the GL and the carbohydrates content as well as carbohydrate-to-DF ratios in vegetables (n=65). **(A)** correlations between the GL versus TC **(B)** correlations between the GL versus DS **(C)** correlations between GL

versus TC-to-DF ratio **(D)** correlations between GL versus DS-to-DF ratio. In all graphs, each data point represents distinct vegetable sample, with GL values on the Y-axis and carbohydrate metrics on the X-axis. Solid lines indicate linear regression trends, reflecting the strength and direction of associations. Selected data points are labeled in grey for emphasis and identification.

Table S4. Statistical analysis of carbohydrate content and GL in various Vegetables (n=65).

Values	Glucose	Total Glucose	Fructose	Total Fructose	Total Sugar	Total Carbohydrates	Dietary Starch	Sucrose	Dietary fiber
R	-0.1859	-0.1414	-0.2402	-0.1905	-0.1288	0.2937	0.2519	0.01528	0.2483
R^2	0.03456	0.01999	0.05769	0.03629	0.0166	0.08624	0.06344	0.00023	0.06163
p-values	0.1382	0.2613	0.0540	0.1285	0.3064	0.0176	0.0447	0.9038	0.0462

Table S5. Statistical analysis of individual carbohydrates-to-dietary fiber ratio and GL in various vegetables (n = 65).

Values	Glucose	Total Glucose	Fructose	Total Fructose	Total Sugar	Total Carbohydrates	Dietary Starch	Sucrose
R	-0.2152	-0.2144	-0.2134	-0.2140	-0.1981	0.1229	0.1604	-0.006926
R^2	0.04630	0.04598	0.04553	0.04580	0.03925	0.01510	0.02574	4.797e-005
p-values	0.0852	0.0863	0.0879	0.0869	0.1136	0.3294	0.2017	0.9563

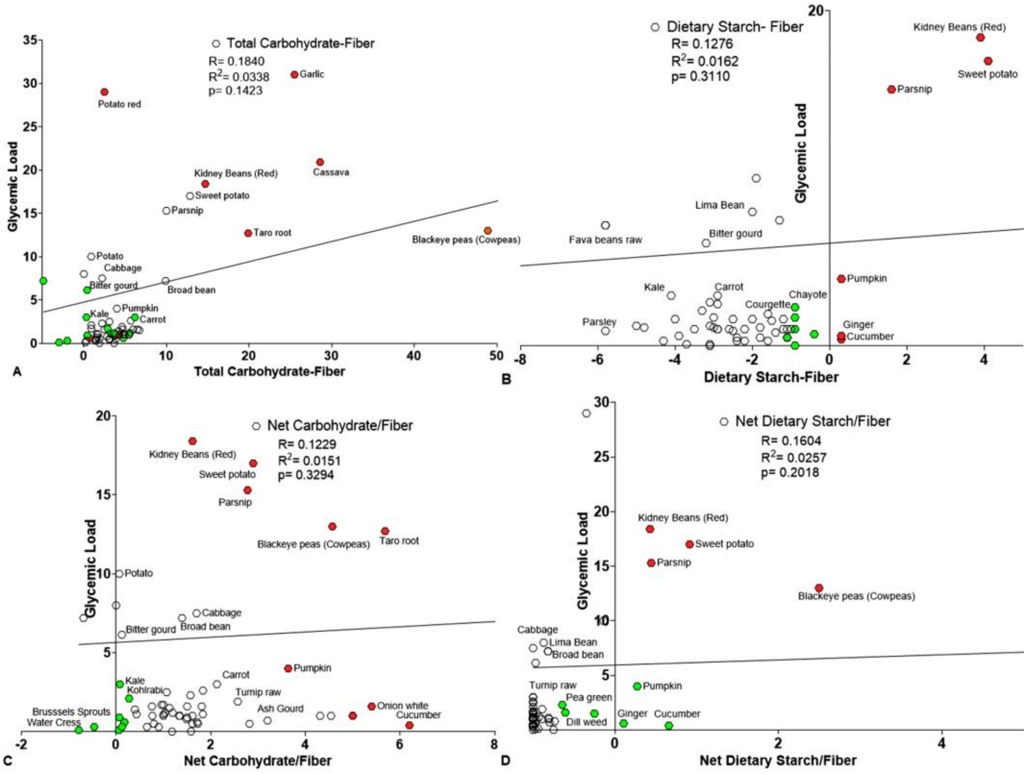

Figure S3. A-D: The correlation plot illustrating the relationship between the GL and vegetable derive available carbohydrates content as well as available carbohydrate-to-DF ratios in vegetables (n=65). **(A)** correlations between the GL versus available TC **(B)** correlations between the GL versus available DS **(C)** correlations between GL versus available TC-to-DF ratio **(D)** correlations between GL versus available DS-to-DF ratio. In all graphs, each data point represents distinct vegetable sample, with GL values on the Y-axis and carbohydrate metrics on the X-axis. Solid lines indicate

linear regression trends, reflecting the strength and direction of associations. Selected data points are labeled in grey for emphasis and identification.

Table S6. Statistical analysis of individual net available carbohydrate and GL in various vegetables (n=65).

Values	Glucose	Total Glucose	Fructose	Total Fructose	Total Sugar	Total Carbohydrates	Dietary Starch	Sucrose
R	-0.4985	-0.4969	-0.5080	-0.5043	-0.4736	0.1840	0.1276	-0.4840
R^2	0.2485	0.2469	0.2558	0.2543	0.2243	0.03387	0.01629	0.2343
p-values	<0.0001	<0.0001	<0.0001	<0.0001	<0.0001	0.1423	0.3110	<0.0001

Table S7. Statistical analysis of available carbohydrate content-to-fiber ratio and GL in various vegetables (n=65).

Values	Glucose	Total Glucose	Fructose	Total Fructose	Total Sugar	Total Carbohydrates	Dietary Starch	Sucrose
R	-0.2152	-0.2144	-0.2134	-0.2140	-0.1981	0.1229	0.1604	-0.006889
R^2	0.04630	0.04598	0.04553	0.04580	0.03925	0.01510	0.02573	4.746e-005
p-values	0.0852	0.0863	0.0879	0.0869	0.1136	0.3294	0.2018	0.9566
